# Targeted peptide modification of mesenchymal stem cells enhances their therapeutic efficacy in the treatment of idiopathic pulmonary fibrosis

**DOI:** 10.3389/fcell.2025.1710750

**Published:** 2025-12-18

**Authors:** Zelin Lei, Rui Jia, Yixuan Ren, Yirun Zhao, Yun Wang, Wenqian Cao, Xue Bai, Yali Peng

**Affiliations:** 1 Lanzhou University First Hospital, Lanzhou, China; 2 Lanzhou University, Lanzhou, China; 3 Gansu University of Chinese Medicine, Lanzhou, China

**Keywords:** mesenchymal stem cells, lung targeted peptide, cell surface modification, stem cell therapy, idiopathic pulmonary fibrosis

## Abstract

**Background:**

Mesenchymal Stem Cells (MSCs), derived from the mesoderm, are adult stem cells characterized by self-renewal, multipotency, and low immunogenicity, making them promising candidates for regenerative therapies. Their intrinsic capacity to migrate to sites of injury and differentiate into diverse cell types presents considerable therapeutic potential. Particularly for lung diseases such as Idiopathic Pulmonary Fibrosis (IPF)—a chronic, progressive, and fatal lung condition with limited treatment options. Despite the potential of MSCs therapy, key challenges remain, including poor homing efficiency and limited retention in target tissues, particularly after systemic administration. Current methods do not adequately address these limitations, resulting in suboptimal therapeutic outcomes in IPF treatment. Enhancing the homing and retention of MSCs in lung tissue is critical for maximizing their therapeutic efficacy, yet an effective strategy for overcoming this challenge is still lacking.

**Methods:**

Here, the synthesis of SA_2_-PEG-Peptides and their analogs was conducted using solid-phase peptide synthesis (SPPS). Two distinct strategies were devised: the first based on metabolic glycoengineering with *in vivo* bioorthogonal copper-free click chemistry to modify functional molecules on the MSCs surface, and the second involving phospholipid-polyethylene glycol modification of MSCs, coupling lung-targeted peptides with phospholipids for surface modification. The efficacy of these strategies was evaluated by examining retention time on the cell membrane, cell viability, cytotoxicity, membrane integrity, hemolysis, and drug distribution in mice.

**Results:**

While the Metabolic Glycoengineering (MGE) approach did not achieve the desired modification results, the co-modification strategy using SA_2_-PEG_2000_ and SA_2_-PEG_2000_-CAR significantly enhanced the homing and retention of MSCs in lung tissue. This modification also substantially improved the therapeutic efficacy of MSCs in treating IPF.

**Conclusion:**

In this study, we developed a cellular modification strategy based on SA_2_-PEG-Peptides and PEGylation. Co-modifying MSCs with SA_2_-PEG_2000_ and SA_2_-PEG_2000_-CAR markedly improved their lung-targeting and retention capacity, resulting in enhanced therapeutic outcomes for IPF. This strategy offers a potential pathway for optimizing MSCs therapies for lung diseases and may be applied to enhance the efficacy of stem cell therapies across a variety of conditions.

## 1 Introduction

Idiopathic pulmonary fibrosis (IPF), the most common type of fibrotic interstitial lung disease, is a progressive, irreversible, and ultimately fatal disease (Glass et al., 2022; Gunatilaka et al., 2023; Kapoor et al., 2025; Q. Liu et al., 2023; Maher, 2024). It is characterized by aberrant differentiation of myofibroblasts and excessive accumulation of collagen-rich extracellular matrix (ECM) (Pei et al., 2022), leading to structural stiffening of the lungs, formation of fibrotic lesions, and irreversible loss of pulmonary function. Current clinical management strategies for IPF encompass both pharmacological and non-pharmacological approaches (D. H. Kim et al., 2025; Lamb, 2021; Oga et al., 2009). Mesenchymal stem cells (MSCs), pluripotent adult stem cells, can modulate the immune system and promote angiogenesis through differentiation and paracrine mechanisms (K. Liu et al., 2024; Melis et al., 2025; Németh et al., 2009). Their low MHC I expression and lack of MHC II (Hwang et al., 2015), along with the absence of costimulatory molecules like CD80, CD40, and CD86, endow them with immune-evasive properties and low immunogenicity (Chan et al., 2006). Notably, the lung may provide a unique niche for MSCs, making MSC-based therapies increasingly used for lung diseases (L.-T. Wang et al., 2016). Despite their broad therapeutic potential shown in numerous studies, challenges remain. As demonstrated in the study by Németh et al., intravenously administered murine MSCs accumulate in the lungs, with over half being rapidly taken up by pulmonary macrophages (Németh et al., 2009; Ouji-Sageshima et al., 2024). The surviving few move to organs like the liver and spleen before being cleared. This leads to inadequate homing and significant loss of MSCs reaching the target site, often necessitating higher doses for effective treatment (Jingxuan et al., 2023; Tian et al., 2021; Zhao et al., 2020).

To address this challenge, the present study engineered the surfaces of MSCs by conjugating targeting peptides to enhance their retention at pathological sites. Two distinct strategies for cell surface modification were employed: (1) covalent coupling via metabolic glycoengineering (MGE)-enabled bioorthogonal reactions (Kufleitner et al., 2023; D. Y. Lee et al., 2018; N. S. Liao et al., 2021), and (2) hydrophobic insertion mediated by lipid-anchored polyethylene glycol (lipid-PEG) derivatives (Kim and Kim, 2022). The MGE approach leverages the metabolic incorporation of azide-functionalized monosaccharides (e.g., Ac_4_ManNAz) into the cellular glycocalyx, thereby generating exogenous azide moieties that serve as bioorthogonal chemical handles (Gutmann et al., 2019; Luchini and Vitiello, 2021). Subsequent copper-free click chemistry between cell-surface azides and dibenzocyclooctyne (DBCO)-functionalized peptides enables site-specific labeling and covalent modification (Lim et al., 2021). The hydrophobic insertion strategy utilizes lipid-PEG conjugates containing terminal maleimide groups (Hulugalla et al., 2024; Takayama et al., 2023). These amphiphilic molecules spontaneously integrate into the lipid bilayer via their hydrophobic domains, while exposing reactive maleimide groups for thiol-mediated peptide conjugation. This membrane-anchoring approach preserves native cell membrane fluidity while providing stable peptide presentation (D. Y. Lee et al., 2018).

THA is a peptide capable of targeting human airway epithelial cells. Jost et al. screened a peptide sequence, H-Thr-His-Ala-Leu-Trp-His-Thr-OH (THALWHT, abbreviated as THA) through phage screening for its ability to target human airway epithelial cells (Jost et al., 2001). A complex comprising the THA sequence and cationic DNA demonstrated efficient gene delivery to human bronchial epithelial cell indicating that the THALWHT peptide can effectively mediate *in vivo* targeting of human airway epithelial cells. To identify peptides capable of homing to neovasculature in healing tissues, Järvinen et al. performed *in vivo* phage display screening using tendon wound models (Jarvinen and Ruoslahti, 2007). This approach led to the identification of a cyclic peptide, H-Cys-Ala-Arg-Ser-Lys-Asn-Lys-Asp-Cys-OH (CARSKNKDC, abbreviated as CAR), which exhibited distinct vascular homing properties (Toba et al., 2014). Intravenous administration of the CAR peptide resulted in significant tissue penetration and preferential accumulation within the granulation tissue of wound beds. Notably, systemic delivery of CAR showed dual targeting specificity, with significant accumulation in both healing wounds and pulmonary vasculature (Maldonado et al., 2023). In addition, modification of the delivery system using stearic acid (SA) enhances anchoring of the complexes on the surface of MSCs(Li et al., 2024).

This study aimed to enhance the homing and retention capabilities of MSCs in lung tissues following systemic administration via surface modification in mice. We developed a cellular modification strategy using SA_2_-PEG-peptides and PEGylation. Co-modification with SA_2_-PEG_2000_ and SA_2_-PEG_2000_-CAR significantly enhanced MSC homing and retention within lung tissue, thereby improving their therapeutic efficacy in IPF.

## 2 Materials and methods

### 2.1 Cell lines

C57BL/6 mouse bone marrow mesenchymal stem cells (abbreviated in the text as BMSCs) were sourced from Cyagen Biosciences Inc. (United States) and MSCs were grown in C57BL/6 mouse bone marrow mesenchymal stem cell medium (Cyagen Biosciences Inc., United States) supplemented with 10% fetal bovine serum (FBS) (Cyagen Biosciences Inc., United States) at 5% CO_2_ and 37 °C.

### 2.2 Animals

The animals used in this experiment were purchased from the Animal Experiment Center of Lanzhou University. The strains included 6–8-weeks-old Kunming mice and C57BL/6 mice. The mice were housed in a dedicated animal room under the following conditions: temperature of 22 °C ± 2 °C, a 12-h light/dark cycle, and were fed specialized breeding mouse food. The animal experiments were approved by the Ethics Committee of the School of Life Sciences, Lanzhou University, Lanzhou, China, under approval number EAF2020022.

### 2.3 Peptide synthesis and purification

The peptides and their analogs used in this study were synthesized step-by-step using the Fmoc-protected strategy and solid-phase peptide synthesis (SPPS) technique. The names and sequences of the targeted peptides synthesized in the experiment are shown in Supplementary Table S1. Additionally, the coupling of lipids to the target peptides was achieved by SPPS using either 2-chlorotrityl (2-Cl-Trt) resin or Wang resin, with 5-carboxyfluorescein (5-FAM) serving as a fluorescent probe attached to the peptide side chains. Purification: The peptide was loaded onto a C8 reverse-phase column (20 × 250 mm, 10 µm), operating on an HPLCONE. Samples were eluted with solvent A (40% isopropanol, 54% acetonitrile, and 6% H_2_O, supplemented with 0.1% TFA) and solvent B (H_2_O supplemented with 0.1% TFA) at a flow rate of 10 mL/min. The gradient was programmed as follows: 0–30 min, 10%–80% solvent A; 30–50 min, 80%–100% solvent A; 50–60 min, 100% solvent A. UV detection was performed at wavelengths of 220 nm and 254 nm.

Purity analysis: The peptide was loaded onto a C8 reverse-phase column (4.6 × 250 mm, 5 µm), analyzed using a Hanbon Sci. & Tech HPLC. Samples were eluted with solvent A (40% isopropanol, 54% acetonitrile, and 6% H_2_O, supplemented with 0.1% TFA) and solvent B (H_2_O supplemented with 0.1% TFA) at a flow rate of 1 mL/min. The gradient program was as follows: 0–10 min, 10% solvent A; 10–30 min, a linear increase from 10% to 100% solvent A; and 30–40 min, 100% solvent A. UV detection was performed at wavelengths of 220 nm and 254 nm.

### 2.4 Determination of the critical micelle concentration (CMC)

The master mix of lipid-peptide conjugates was diluted with PBS to final concentrations of 0, 1, 5, 10, 20, 30, 50, and 100 μM, followed by centrifugation with shaking and incubation at 37 °C for 2 h. Thereafter, 0.04 μM concentration of the ANS-Na fluorescent probe was then added to each sample group, followed by centrifugation with shaking and incubation at 37 °C for 20 min. The optical density (O.D.) of each sample in PBS buffer was measured using a fluorescence spectrophotometer at an excitation wavelength of 330 nm and an emission wavelength of 492 nm. Linear regression analysis was conducted on the plot of measured O.D. values against protein concentrations to determine the critical micelle concentration (CMC) of the lipid-peptide complexes.

### 2.5 Ac_4_ManNAz cell incubation and bioorthogonal reactions of DBCO analogs

Ac_4_ManNAz was diluted to experiment-specific concentrations using the medium supplemented with 10% fetal bovine serum. The original medium in the Petri dish was aspirated, followed by washing with D-PBS. The prepared Ac_4_ManNAz dilution was then added, mixed thoroughly, and incubated in a CO_2_ incubator at 37 °C with 5% CO_2_ and saturated humidity for 72 h. After incubation, the Ac_4_ManNAz solution was aspirated, and the cells were washed three times with D-PBS. The basal medium was then combined with the DBCO (Lu et al., 2025) analog at experiment-specific concentration, mixed thoroughly, and added to the Petri dishes for further incubation. After the specified incubation period, the medium was aspirated, the cells were washed three times with D-PBS, and the medium was replaced with complete medium.

### 2.6 Cell membrane co-localization

The lyophilized Lipid-PEG-Peptides powder was dissolved in a mixture of DMSO and water at a specified ratio to prepare a 1 mM stock solution. This stock solution was then diluted to various concentrations using culture medium, and the cells were co-incubated with the peptide drug for a predetermined duration. Subsequently, the cells were washed three times with D-PBS and incubated with 10 μg/mL of the cytosolic dye Hoechst 33342 (Beyotime, China) for 10 min at a density of 2.4 × 10^5^ cells per well. Following incubation, the cells were washed three additional times with D-PBS to remove excess dye and imaged using a high-content imaging system (ImageXpress, Molecular Devices, United States) under culture medium conditions to assess peptide drug membrane incorporation.

### 2.7 Trypan blue (TB) staining assay

The drug master mix was diluted to various concentrations and co-incubated with 2.4 × 10^5^ cells per well. After a specific incubation period, TB was added to quench the external fluorescence of the cell membrane. Subsequently, the dye was then washed out with PBS, and the average fluorescence intensity of the samples was measured using the FITC channel of a flow cytometer (NovoCyte Quanteon, Agilent, United States).

### 2.8 Cell counting kit-8 (CCK-8) assay

After seeding 5,000 cells suspended in 100 μL were seeded into each well of a 96-well plate, which was then pre-incubated for 24 h in a humidified incubator maintained at 37 °C with 5% CO_2_. Subsequently, 10 μL of various concentrations of the test substances were added to each well, and the plate was incubated for the designated duration (24, 48, 72, or 96 h). Following incubation, 10 μL of CCK-8 solution (Med Chem Express, United States) was carefully added to each well, avoiding bubble formation to prevent interference with the O.D. readings. The plate was then incubated for an additional 1–4 h before absorbance was measured at 450 nm using a microplate reader. Each experimental group included three replicate wells.

$$\text{Cytotoxicity}=\frac{\text{As}-\text{Ab}}{\text{Ac}-\text{Ab}}\times 100\%$$

As: absorbance of experimental wells containing cells, culture medium, CCK-8 solution, and drug solution;

Ac: absorbance of the control well (containing cells, culture medium, and CCK-8 solution, but without drug solution);

Ab: absorbance of the blank control (containing cells and CCK-8 solution, but without the drug solution).

This procedure also detects cell proliferation.

### 2.9 Apoptosis assay

Cells were resuspended in 1 mL of the drug solution and incubated on a shaker at 37 °C and 300 rpm. After incubation, the cells were washed three times with PBS and then resuspended in 100 μL of 1× Binding Buffer. The cell suspension was transferred to flow cytometry tubes, and 5 μL of YF®488-Annexin V (Shanghai BioScience, China) and 5 μL of PI working solution were added to each tube. The samples were incubated at room temperature in the dark for 10–15 min. Subsequently, the cells were resuspended in 400 μL of 1× binding buffer per tube and analyzed using the FITC/PI channel of a flow cytometer.

Treatment group: 20 μM SA_2_-(PEG_2_)_2_-THA and 50 μM SA_2_-PEG_2000_-CAR.

Negative control group: untreated cell samples.

### 2.10 Measurement of cell membrane integrity via lactate dehydrogenase (LDH) assay

The master drug solution was diluted to final concentrations of 0, 1, 5, 10, 20, 30, 50, and 100 μM. Cells were then incubated with the drug at 37 °C for 2 h. Following incubation, the samples were collected and centrifuged. From each sample, 120 μL of the supernatant was carefully aspirated and mixed with 60 μL of the working solution from the LDH Cytotoxicity Assay Kit (Beyotime, China). The mixture was incubated on a shaking platform at room temperature, protected from light, for 30 min. O.D._490_ was measured using a Thermo Varioskan Flash multifunctional enzyme reader (Thermo Scientific, United States). Each experimental group was set up with 3 duplicate wells, and the experiments were independently repeated three times.

Blank control: cell-free medium;

Control: cell wells without drug;

Cell maximum enzyme activity: cell wells with 10% volume of LDH release reagent (added 1 h before the assay and mixed thoroughly by repeated pipetting).

Treated: cell wells with prepared samples of different concentrations.

$$\text{LDH Release}=\frac{\text{Ad}-\text{Ab}}{\text{Ac}-\text{Ab}}\times 100\%$$

Aa: absorbance of blank;

Ab: absorbance of control;

Ac: absorbance of cell maximum enzyme activity;

Ad: absorbance of treatment.

### 2.11 Hemolytic activity assay

Prepare a 2 mg/mL solution of heparin sodium (Sigma-Aldrich, Germany) in PBS. Add 150 μL of the prepared heparin sodium solution to a 1.5 mL centrifuge tube. Select Kunming mice weighing 18–25 g and collect approximately 1 mL of fresh blood from the eyeball, then add it to the pre-cooled centrifuge tube containing sodium heparin. Mix the blood and sodium heparin by gently inverting the tube to prevent clotting. Centrifuge at 4 °C, 800 g for 10 min and discard the supernatant. Resuspend the cells by adding 1 mL of PBS, centrifuge again, discard the supernatant, and repeat this washing step three times. The resulting erythrocytes are considered as 100% and diluted to 8% of the total volume with PBS. Add 100 μL of the diluted erythrocytes to each well of a 96-well plate. Prepare the test samples as dilutions of 0, 1, 5, 10, 20, 30, 50, and 100 μM in basal medium. The samples were incubated at 37 °C for 1 h. After incubation, centrifuge the plate at 4 °C at 1,000 g for 15 min. Carefully transfer 150 μL of the supernatant from each well to a new 96-well plate. Measure the absorbance of each well at 490 nm using a Thermo multi-functional enzyme marker. Each experimental group was set up in triplicate wells, and three independent replicate experiments were performed in total.

Treat: cell wells with prepared samples of different concentrations;

Negative control: cell wells with 100 μL of PBS buffer;

Positive control: cell wells with 100 μL of PBS buffer containing 4% Triton X-100.

$$\text{Hemolytic activity}=\frac{\text{As}-\text{An}}{\text{Ap}-\text{An}}\times 100\%$$

As: absorbance of treatment;

An: absorbance of negative control;

Ap: absorbance of positive control.

### 2.12 Cell adhesion assay

Cell adhesion assays were performed in 24- plates coated with collagen I (CI) at a concentration of 5 μg/cm^2^ (Ji et al., 2024). The wells were washed 3 times with PBS and blocked with 1% bovine serum albumin (BSA) in PBS for 1 h at 37 °C. MSCs were preincubated with SA_2_-PEG_2000_-CAR and SA_2_-(PEG_2_)_2_-THA at 50 μM for 30 min. Cell-free medium served as a blank, and unmodified MSCs were used as a control. Then 3 × 10^4^ MSCs were seeded into each well and incubated for 30 min at 37 °C. The MSCs were subsequently washed three times with PBS and resuspended in serum-free medium (Honig and Shapiro, 2020). The number of adherent cells was determined using the Cell Counting Kit-8 (Med Chem Express, United States), with n = 4.

### 2.13 Construction and administration of bleomycin (BLM) mouse lung fibrosis model

C57BL/6 mice weighing 20–25 g were selected for the lung fibrosis model. The mice were anesthetized with 1.25% Tribromoethanol (sigma) at a dosage of 250 mg/kg. Following sterilization of the neck area with 75% alcohol, the mice were placed in a supine position on a mouse board. A 1 cm incision was made in the neck, and the trachea was exposed via blunt dissection. Using a 1 mL syringe, BLM was administered via tracheal instillation at a dose of 3 mg/kg (Chen et al., 2024) in a total volume of 100 μL. After completing the administration, the incision was closed. For the control group of the IPF model, 100 μL of PBS buffer was injected into the trachea after anesthesia.

Seven days following the administration of bleomycin via tracheal drip, tail vein injections of PBS, MSCs, CAR-MSCs, and PCAR-MSCs were initiated. MSCs for each experimental group were resuspended in a 1:1 ratio of 1 mg/mL sodium heparin solution and PBS buffer, making up a total volume of 200 μL. MSCs were administered every 7 days, with 1 × 10^6^ cells in each injection, for a total of three administrations. After 28 days, the mice were sacrificed, and lung tissue was collected for Masson staining and H&E staining.

### 2.14 *In vivo* targeting assay

Male Kunming mice weighing 22–25 g were selected for *in vivo* targeting experiments. Cells were labeled with the lipophilic fluorescent dye (DID, Solarbio, China), which is suitable for *in vivo* tracer studies. MSCs were incubated with 30 μM DID for 2 h. After three washes with PBS, the cells were digested with trypsin. SA_2_-PEG_2000_-CAR solution was prepared in basal medium and added to the MSCs separately, ensuring the cells were gently agitated to allow thorough contact with the solution. The cells were incubated on a shaker for 15, 30, and 30 min, respectively. Following incubation, the cells were centrifuged at 1,500 rpm for 5 min, and the supernatant was discarded. MSCs, CAR-PEG_2000_-MSCs, and PCAR-PEG_2000_-MSCs were obtained. The cells were resuspended in 1 mL of PBS, centrifuged, and the supernatant was discarded. This process was repeated three times. After washing, the cells were counted and resuspended in PBS containing 0.5 mg/mL sodium heparin, followed by gentle agitation. A single-cell suspension containing 1 × 10^6^ cells per 200 μL of PBS was prepared. According to the time gradient, 200 μL of the peptide-modified MSCs suspension was injected into the tail vein of different groups of mice at 0.5, 6, 12, 24, 48, 72, and 96 h before the assay. Non-drug-treated MSCs served as the control group, and PBS alone was injected as the blank control. After injection, the mice were housed in cages for further observation.

The mice in the 0.5, 6, 12, 24, 48, 72, and 96 h post-injection groups were euthanized, and major organs (heart, liver, spleen, lungs, and kidneys) were harvested. The distribution of MSCs was observed and quantitatively analyzed using a Bioin vivo imaging system (Aniview100, Guangzhou Biolight Biotechnology Co. Ltd., China) at Ex/Em: 644/663 nm.

### 2.15 Quantitative reverse transcription PCR

Total RNA was extracted from mouse lung tissue using the SteadyPure Universal RNA Extraction Kit II (Accurate Biotechnology, China). 1 μg of total RNA was used for the reverse transcription of RNA into cDNA in a reaction using the Evo M-MLV RT Mix Kit (Accurate Biotechnology, China). SYBR Green Premix Pro Taq HS qPCR Kit (Accurate Biotechnology, China) was used, and relative gene expression quantitation was determined using the 2^−ΔΔCT^ method and normalized to the *GAPDH* gene. There were three replicates per group. The PCR primers are listed in Supplementary Table S3 (Beijing Tsingke Biotech, China). Fluorescence signals were detected by Mx3005P Real-Time PCR System (Agilent Technologies Inc., United States).

## 3 Results

### 3.1 FAM-Lys (DBCO)-THA modified BMSCs via MGE and copper-free orthogonal click chemistry techniques

Initially, SPPS was used to synthesize the DBCO-targeted peptide conjugate, followed by fluorescent labeling with the dye 5-FAM, yielding FAM-K (DBCO)-THA. Both RT-HPLC analysis and ESI-MS confirmed that the purity of FAM-K (DBCO)-THA exceeded 95% (Supplementary Table S2). Additionally, laser confocal microscopy demonstrated successful stem cell modification based on MGE and bioorthogonal click chemistry (Supplementary Figure S1). Furthermore, we demonstrated that azide groups generated on the cell surface were saturated at a 20 μM Ac_4_ManNAz incubation, and a concentration-dependent reaction occurred between sulfo-Cy5.5-DBCO and the azide groups (Supplementary Figures S2A,B). Moreover, incubation time had no significant effect on either experimental set (data not shown).

### 3.2 Optimisation of conditions for FAM-THA modification

The targeted peptide THA was conjugated to DBCO, forming THA-DBCO. Co-localization results (Supplementary Figure S3A) indicated that the red fluorescence of sulfo-Cy5.5-DBCO overlapped with the green fluorescence of FAM carried by FAM- THA, producing distinct yellow fluorescence spots, confirming successful co-localization. The group treated with free THA did not display visible FAM fluorescence, suggesting that FAM-THA effectively modified MSCs through the reaction between DBCO in the coupler and azide groups generated on the cell membrane. To optimize stem cell modification efficiency using metabolic glycan engineering and bioorthogonal click chemistry, the incubation time and concentration of FAM-THA were optimized. As shown in Supplementary Figures S3B,C, incubating with 50 μM FAM--THA for 1 h was optimal for targeting cell membranes.

### 3.3 Insufficient retention time of FAM-Lys (DBCO)-THA modification on the cell membrane

The dynamics of membrane flow and flipping may have led to the internalization or detachment of FAM-Lys (DBCO)-THA from the cell membrane. MSCs pretreated with 20 μM Ac_4_ManNAz for 72 h were subsequently incubated with 50 μM Sulfo-Cy5.5-DBCO for 1 h, and fluorescence changed on the cells were observed using microscopic imaging. As shown in Supplementary Figure S4, fluorescence on the cells decreased rapidly over time, with a significant reduction at 12 h and complete loss of detectability at 24 h. These results suggest that metabolic glycan-based engineering and bioconjugation of MSCs are applicable for MSC treatments. However, the cellular modification approach using metabolic glycan engineering and bioorthogonal click chemistry exhibited insufficient retention time to effectively enhance the lung-targeting ability of MSCs.

### 3.4 SA_2_-FAM modified MSCs and the effect of drug incubation duration on internalization

Previous studies on MGE-based stem cell targeting modifications did not show the expected results. Therefore, this study attempted to explore alternative cell surface modification to conjugate targeted peptides onto stem cells (Kim and Kim, 2022; Zhong et al., 2021). SPPS was used to synthesize SA_2_-PEG-Peptides, and their quality was assessed by RT-HPLC (Supplementary Table S2). The results demonstrated that the purity of the targeted peptides and their analogs exceeded 95%, and mass spectrometry results aligned with the calculated theoretical values.

**FIGURE 1:**
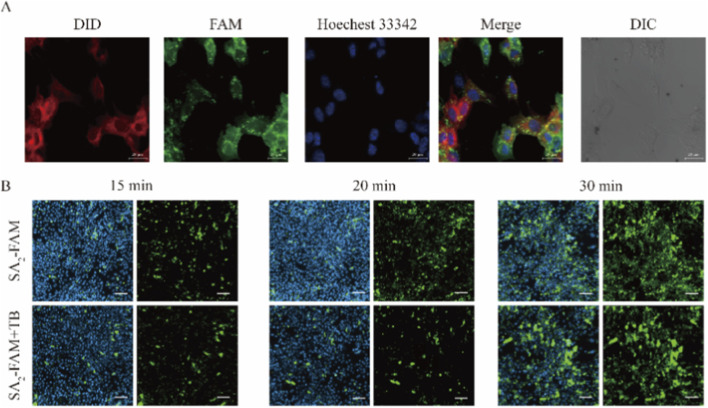
SA_2_-FAM co-localized with cell membranes, modification time and cell internalization. **(A)** SA_2_-FAM (10 μM) was incubated for 1 h, followed by washing and staining with the cell membrane dye DID (10 μM) for 1 h. Fluorescence co-localization was observed by laser confocal, red: DID dye; green: SA_2_-FAM; blue: Hoechst 33342. Scale bar: 20 μm. **(B)** 10 μM SA_2_-FAM was incubated for 15, 20, and 30 min 1 mg/mL of Trypan blue (TB) was added to quench the fluorescence on the cell membranes, and the cells were washed after 2 min of incubation. High-content cell imager observation. Green: SA_2_-FAM; blue: Hoechst 33342. Scale bar: 20 μm.

We utilized a stearic acid double chain (SA_2_) resembling lipid and incorporated it into the mesenchymal stem cell membrane through hydrophobic interactions, subsequently enabling targeted delivery of MSCs with the SA_2_ targeting peptide. Therefore, the SA_2_ modification needed to be retained in the cell membrane to expose the attached targeting peptide on the cell surface. MSCs were incubated with the cell membrane fluorescent probe DID and SA_2_-FAM. As shown in Figure 1A, both DID and FAM fluorescence were distributed around the MSCs, and the green fluorescence (SA_2_-FAM) overlapped with the red fluorescence (DID), producing a distinct yellow fluorescent signal. This indicates that SA_2_-FAM successfully modified the MSC cell membrane.

Due to the motility of the cell membrane, SA_2_-FAM, which was inserted on the membrane surface, was endocytosed by the cell, preventing the drug from remaining exclusively on the membrane surface. To prolong the retention of SA_2_-FAM on the cell membrane, we observed the effects of drug-cell co-incubation at various time points. The fluorescence on the cells gradually increased as the incubation time extended (Figure 1B, top), indicating that the amount of SA_2_-FAM inserted into the MSC membranes increased with longer incubation. As shown in Figure 1B (bottom), fluorescence was largely quenched after 15 and 20 min, suggesting that the majority of SA2-FAM remained on the cell surface, with only a minor fraction internalized. When the incubation time was extended to 30 min, some fluorescence was still retained in the MSCs, indicating that SA_2_-FAM was inserted into the cell membrane and underwent cellular internalization. Thus, by controlling the incubation time, it is possible to achieve high-efficiency membrane surface modification with the drug.

### 3.5 Construction of SA_2_-PEG-peptide complexes for the modification of stem cell membranes

**FIGURE 2:**
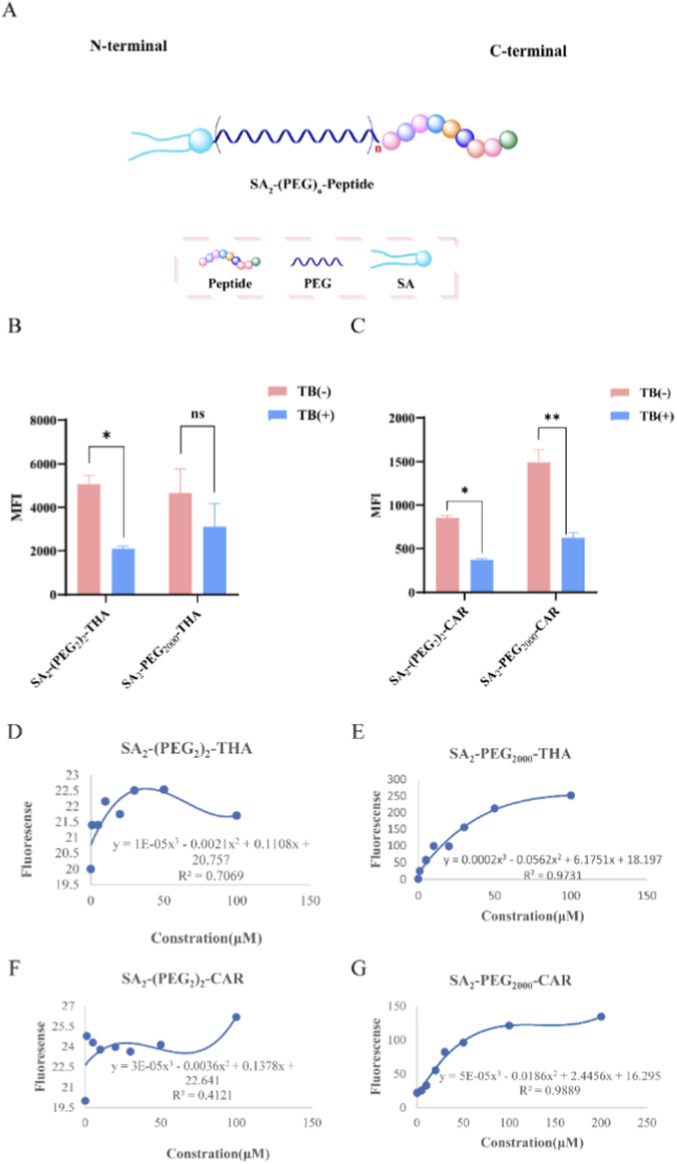
Efficiency of cell membrane modification by SA_2_-(PEG)n-Peptides and their analogs. **(A)** Schematic design of SA_2_-(PEG)n-Targeted peptide analogs. The phospholipid portion is more hydrophobic and the targeting peptide and modified polyethylene glycol portion are more hydrophilic. **(B)** THA analogs: 5-FAM-labeled SA_2_-(PEG)n -THA analogs were added to MSCs for incubation. SA_2_-(PEG_2_)_2_-THA, SA_2_-PEG_2000_-THA 20 μM incubation for 15 min **(C)** CAR analogs: SA_2_-(PEG_2_)_2_-CAR, SA_2_-PEG_2000_-CAR 20 μM incubation for 15 min Quenching group: 1 mg/mL Trypan blue was added to quench the extracellular fluorescence for 2 min. Flow cytometry was used to detect the total fluorescence intensity on the cells and the fluorescence intensity that was internalized by the cells. **(D–G)** The critical micelle concentration of each phospholipid-targeting peptide was calculated by taking the absorbance curve with concentration as the variable and finding the regression equation of the curve. SA_2_-(PEG_2_)_2_-THA, SA_2_-PEG_2000_-THA, SA_2_-(PEG_2_)_2_-CAR, SA_2_-PEG_2000_-CAR, had critical micelle concentrations of 70, 181, 45 and 113.1 μM, respectively. n = 3. *p < 0.05; **p < 0.01; ****p < 0.0001 indicating that this experimental group showed a Statistical difference.

The strong hydrophobicity of SA combined with the hydrophilicity of PEG-conjugated targeting peptides significantly reduces the internalization of SA_2_-PEG-Peptides during the modification process. To enhance cell membrane modification efficiency and targeting capability, various Lipid-PEG-Peptides were synthesized and differentially modified based on three targeting peptides, including THA and CAR, in this study. As illustrated in Figure 2A, SA were conjugated to either the N- or C-terminus of each targeting peptide to improve the insertion efficiency of the compounds into the cell membrane. Additionally, PEG chains of varying lengths were introduced between the SA and targeting peptides to modulate cellular internalization. However, owing to the amphiphilic properties of these analogs, micelle formation occurred at certain concentrations, which adversely affected the efficiency of SA insertion into MSC membranes. Hydrophobic fluorescent probes (ANS-Na) were used to determine the critical micelle concentration (CMC) of each group of modified peptides. As illustrated in Figures 2D–G, the peptides SA_2_-(PEG_2_)_2_-THA, SA_2_-PEG_2000_-THA, SA_2_-(PEG_2_)_2_-CAR and SA_2_-PEG_2000_- CAR exhibited distinct CMC values; however, none formed significant micelles at higher concentrations, indicating that micelle formation did not compromise membrane modification efficiency. Further modifications of the SA_2_-PEG peptides involved variations in the SA linkage site and PEG chain length. These analogs were labeled with the fluorescent dye 5-FAM to facilitate comparative analysis. The membrane insertion efficiencies of the different analogs were assessed through quantitative flow cytometry following treatment with Trypan blue to evaluate fluorescence differences among groups. As shown in Figure 2B, after incubation at 20 μM for 15 min of incubation, the fluorescence intensities corresponding to SA_2_-(PEG_2_)_2_-THA and SA_2_-PEG_2000_-THA accounted for 55.28% and 55.73%, respectively, of the total cellular fluorescence. Similarly, fluorescence associated with SA_2_-(PEG_2_)_2_-CAR and SA_2_-PEG_2000_-CAR cell membranes represented 55.87% and 58.16%, respectively (Figure 2C). These results indicate that SA_2_-PEG_2000_-CAR exhibits superior cell membrane insertion efficiency. Consequently, subsequent experiments were performed using SA_2_-(PEG_2_)_2_-THA and SA_2_-PEG_2000_-CAR.

### 3.6 SA_2_-PEG-peptide complexes exhibit limited cellular internalization

**FIGURE 3:**
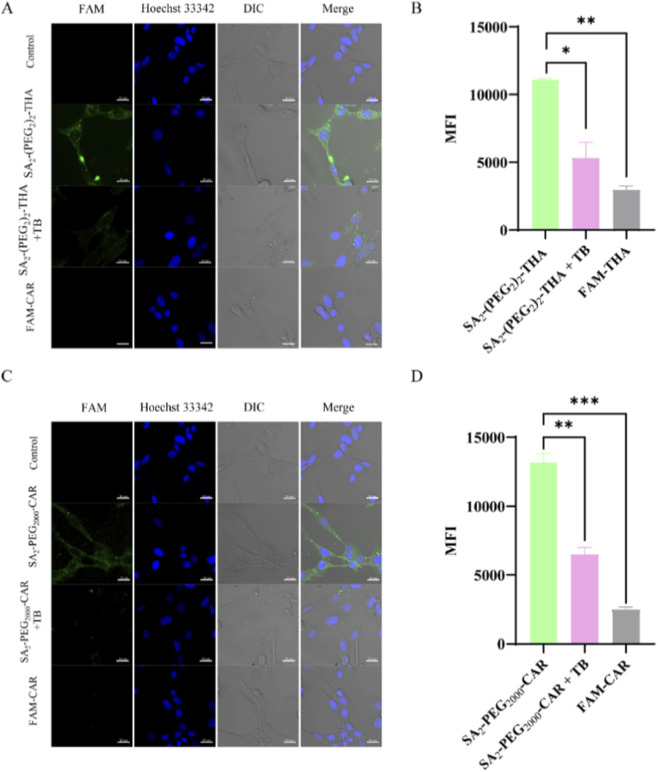
Cell internalization assay. **(A)** SA_2_-(PEG_2_)_2_-THA group: 20 μM SA_2_-(PEG_2_)_2_-THA was incubated for 30 min. Fluorescence on the cell membrane was quenched by the addition of 1 mg/mL of Trypan blue, and the cells were incubated for 2 min and visualized by a high-intensity cell imager. Free THA was used as a control. **(B)** Flow quantification of cell internalization; Green: SA_2_-(PEG_2_)_2_-THA; Blue: Hoechst 33342. Scale bar: 20 μm. **(C)** SA_2_-PEG_2000_-CAR group: 20 μM SA_2_-PEG_2000_-CAR was incubated for 30 min 1 mg/mL Trypan blue was added to quench the fluorescence on the cell membrane, and the cells were incubated for 2 min and observed by a high content cell imager. Free CAR was used as a control. **(D)** Flow quantitative analysis of cell internalization; Green: SA_2_-PEG_2000_-CAR; Blue: Hoechst 33342. Scale bar: 20 μm **p < 0.01; ***p < 0.001; indicated that this experimental group exhibited statistical differences compared with the control group.

It was hypothesized that the incorporation of hydrophilic targeting peptides and PEG could effectively inhibit cellular internalization, thereby enhancing the targeting of MSCs. To evaluate this hypothesis, 5-FAM-labeled SA_2_-(PEG_2_)_2_-THA and SA_2_-PEG_2000_-CAR were co-incubated with MSCs for 30 min at a concentration of 20 μM. Following incubation, cell membrane fluorescence was quenched using 1 mg/mL Trypan blue, and fluorescence changes were observed through microscopy and flow cytometry. Strong fluorescence signals were observed on MSCs in all groups prior to quenching. However, post-quenching, the SA_2_-(PEG_2_)_2_-THA and SA_2_-PEG_2000_-CAR groups demonstrated a significant reduction in fluorescence intensity (Figure 3), indicating successful modification of the cell membrane. In contrast, the two free targeting peptides (THA and CAR) exhibited negligible fluorescence, suggesting that cellular modification by the SA_2_-PEG-Peptides was predominantly mediated through hydrophobic interactions between the SA moiety and the cell membrane (Figures 3B,D).

### 3.7 Prolonged retention of lipid hydrophobic modification modalities within cell membranes

**FIGURE 4:**
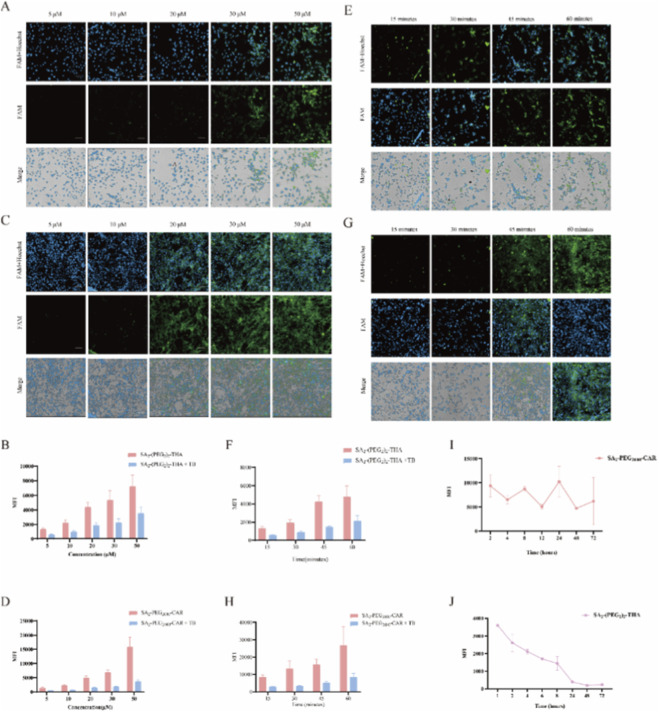
Cell membrane retention time of SA_2_-(PEG_2_)_2_-THA and SA_2_-PEG_2000_-CAR with correct incubation concentration and incubation time. SA_2_-(PEG_2_)_2_-THA group: **(A)** Microscopic imaging of SA_2_-(PEG_2_)_2_-THA incubated for 15 min at 5, 10, 20, 30, and 50 μM. The effect of incubation time on the internalization of SA_2_-(PEG_2_)_2_-THA was detected by quenching the fluorescence on the cell membrane with Trypan blue. **(B)** Results of flow quantitative analysis of cell internalization. Green: SA_2_-PEG_2000_-CAR; blue: Hoechst 33342. n = 3. Scale bar: 50 μm. SA_2_-(PEG_2_)_2_-THA group: **(C)** Microscopic imaging of 5, 10, 20, and 30 μM SA_2_-PEG_2000_-CAR incubated for 30 min. And Trypan blue was added to quench the fluorescence on the cell membrane to detect the effect of incubation concentration on the internalization of SA_2_-PEG_2000_-CAR. **(D)** Results of flow quantitative analysis of cell internalization. Green: SA_2_-PEG_2000_-CAR; blue: Hoechst 33342. n = 3. Scale bar: 50 μm SA_2_-(PEG_2_)_2_-THA group: **(E)** Microscopic imaging of 20 μM SA_2_-(PEG_2_)_2_-THA incubated for 15, 30, 45, and 60 min. The effect of incubation time on SA_2_-(PEG_2_)_2_-THA cell internalization was detected after Trypan blue quenching. **(F)** Results of flow quantitative analysis of cell internalization. Green: SA_2_-(PEG_2_)_2_-THA; blue: Hoechst 33342. n = 3. Scale bar: 50 μm. SA_2_-(PEG_2_)_2_-THA group: **(G)** Microscopic imaging of 20 μM SA_2_-(PEG_2_)_2_-THA incubated for 15, 30, 45, 60 min. The effect of incubation time on SA_2_-(PEG_2_)_2_-THA cell internalization was detected after Trypan blue quenching. **(H)** Results of flow quantitative analysis of cell internalization. Green: SA_2_-(PEG_2_)_2_-THA; blue: Hoechst 33342. n = 3. Scale bar: 50 μm. **(I)** 20 μM SA_2_-(PEG_2_)_2_-THA was incubated for 15 min and quantified by flow cytometry. **(J)** 50 μM SA_2_-PEG_2000_-CAR was incubated for 30 min and quantitatively analyzed by flow cytometry. Quenching group: 1 mg/mL Typan blue quenching for 2 min n = 3.

The targeting efficiency of SA_2_-PEG-Peptides towards MSCs is influenced by the targeting capabilities of the modified peptide, the quantity of peptides successfully conjugated to the cell membrane, and the duration of these modifications on the membrane. To optimize incubation conditions, MSCs were incubated with varying concentrations (5, 10, 20, 30, and 50 μM) of SA_2_-(PEG_2_)_2_-THA and SA_2_-PEG_2000_-CAR for 15 min. Following incubation, peptide fluorescence was quenched using Trypan blue, and the difference in fluorescence before and after quenching was measured to identify the optimal incubation concentration. As shown in Figures 4A,B, the membrane insertion efficiencies of SA_2_-(PEG_2_)_2_-THA were 55.3%, 54.8%, 65.5%, 55.4%, and 45.6% at concentrations of 5, 10, 20, 30, and 50 μM, respectively. These results indicate that 20 μM SA_2_-(PEG_2_)_2_-THA achieved the highest membrane modification efficiency with minimal cellular internalization. A similar analysis of SA_2_-PEG_2000_-CAR revealed insertion efficiencies of 73.84%, 75.04%, 85.55%, 89.62%, and 91.27% at 5, 10, 20, 30, and 50 μM, respectively (Figures 4C,D), suggesting that 50 μM is the optimal concentration for SA_2_-PEG_2000_-CAR surface modification.

Optimal incubation times were evaluated by applying time gradients of 15, 30, 45, and 60 min for each group. The results indicated that the most effective conditions for inhibiting cell membrane internalization20 μM SA_2_-(PEG_2_)_2_-THA with a 15-min incubation and 50 μM SA_2_-PEG_2000_-CAR with a 30-min incubation (Figures 4E–H).

Using these conditions, the retention times of 5-FAM labeled SA_2_-(PEG_2_)_2_-THA and SA_2_-PEG_2000_-CAR on the membranes of MSCs were evaluated via flow cytometry. Over a period of 24 h, the fluorescence intensity of SA_2_-(PEG_2_)_2_-THA gradually diminished (Figure 4I). In contrast, the fluorescence of SA_2_-PEG_2000_-CAR on the cell membranes persisted for up to 72 h without significant reduction, with 66.14% of the fluorescence retained (Figure 4J). These findings suggest that lipid hydrophobic modification, which remains on the cell membrane for an extended duration, may enhance the targeting capability of MSCs relative to MGE modifications.

### 3.8 Biocompatibility assessment of SA_2_-(PEG_2_)_2_-THA and SA_2_-PEG_2000_-CAR demonstrates high compatibility

**FIGURE 5:**
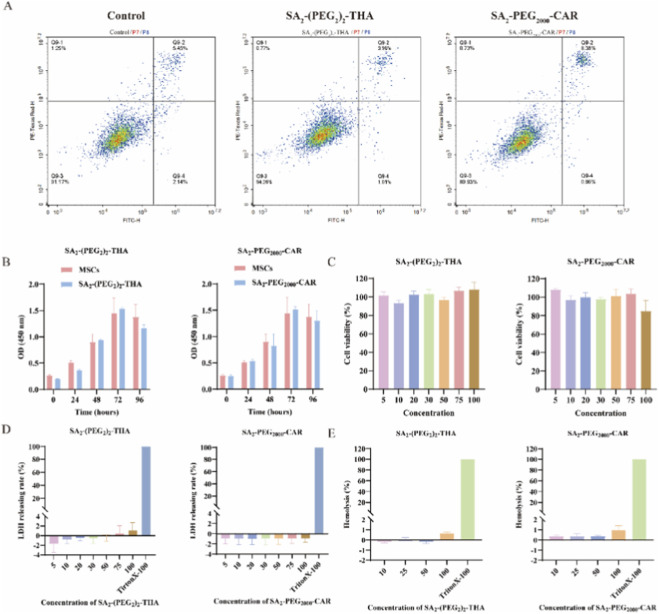
Biosafety Testing of SA_2_-(PEG_2_)_2_-THA and SA_2_-PEG_2000_-CAR. **(A)** Flow cytometry assays were performed using the YF®488-Annexin V/PI Apoptosis Kit. Apoptosis of SA_2_-(PEG_2_)_2_-THA and SA_2_-PEG_2000_-CAR at their respective optimal concentrations was detected by flow cytometry. Left: apoptotic scatter plot of normal cells; Middle: apoptotic scatter plot of SA_2_-(PEG_2_)_2_-THA; Right: apoptotic scatter plot of SA_2_-PEG_2000_-CAR. **(B)** The cell proliferation of three SA_2_-PEG-Peptides: SA_2_-(PEG_2_)_2_-THA (20 μM), SA_2_-PEG_2000_-CAR (50 μM) was determined by CCK-8 kit after incubation for 0, 24, 48, 72, 96 h. The cell proliferation was detected by multifunctional enzyme marker with the OD value of cells at 450 nm, n = 3. **(C)** Cytotoxicity assay. Cytotoxicity of SA_2_-(PEG_2_)_2_-THA and SA_2_-PEG_2000_-CARincubated for 24 h at different concentrations (5, 10, 20, 30, 50, 75, 100 μM). Blade azurite was added and incubated for 2 h. Multifunctional zymography was used to detect the uptake value at 570/590 and calculate the cell survival rate, n = 3. **(D)** LDH assay for cell membrane integrity. Using LDH Cytotoxicty Assay Kit, SA_2_-(PEG_2_)_2_-THA and SA_2_-PEG_2000_-CAR were incubated with MSCs at different concentrations for 2 h. TritonX-100-treated erythrocytes served as a positive control, and cells were detected by multifunctional zymography at OD value at 490 nm and calculated the LDH release rate, n = 3. **(E)** Determination of hemolytic activity of SA_2_-(PEG_2_)_2_-THA and SA_2_-PEG_2000_-CAR erythrocytes. Different concentrations of peptides (10, 25, 50, 100 μM) were incubated with erythrocytes at 37 °C for 1 h. TritonX-100-treated erythrocytes were used as a positive control, and multifunctional enzyme labeling instrument was used to detect the absorption value at 490 and calculate the hemolytic activity at each concentration, n = 3.

Heterozygous, homozygous, and injured MSCs, particularly those exhibiting surface markers such as phosphatidylserine, may trigger physiological tissue clearance mechanisms, leading to immunosuppression or tolerance (Galipeau and Sensebe, 2018). Apoptotic MSCs, which exhibit functional impairment, are cleared *in vivo*, thereby diminishing therapeutic efficacy. To assess whether the hydrophobic insertion of SA_2_-PEG-Peptides induces programmed cell death in MSCs, flow cytometric analysis was performed using the YF®488-Annexin V/PI Apoptosis Kit (Beyotime, China). MSCs were incubated with SA_2_-(PEG_2_)_2_-THA and DSPE-PEG_2000_-CAR at 37 °C under optimal conditions, with untreated MSCs serving as a control. The flow cytometry data were divided into four quadrants: Q1 (necrotic cells), Q2 (late apoptotic cells), Q3 (early apoptotic cells), and Q4 (normal cells). Apoptotic cells were quantified as the sum of Q2 and Q3. As shown in Figure 5A, the control group exhibited 7.59% apoptotic cells, whereas treatment with 50 μM SA_2_-(PEG_2_)_2_-THA for 15 min resulted in 4.97% apoptosis, indicating no significant induction of apoptosis. Conversely, MSCs treated with 50 μM SA_2_-PEG_2000_-CAR for 30 min demonstrated an apoptosis rate of 9.34%. These findings suggest that modifications with both SA_2_-(PEG_2_)_2_-THA and SA_2_-PEG_2000_-CAR maintain high cellular safety.

To evaluate the impact of SA_2_-PEG-Peptides hydrophobic insertion on the proliferation of MSCs, a CCK-8 assay was performed. MSCs were incubated with SA_2_-(PEG_2_)_2_-THA (20 μM for 15 min) and SA_2_-PEG_2000_-CAR (50 μM for 30 min), and their proliferation was monitored over a 96-h period. Figure 5B provides a clear representation of the absorbance values measured at 450 nm for both control and treated groups. Absorbance increased over time in all groups, indicating normal cell proliferation. No significant decrease in absorbance values was observed in any treated group compared to the control within the 96-h timeframe, suggesting that the hydrophobic modification of MSC membranes by the three SA_2_-PEG-Peptides did not adversely affect cell proliferation.

The safety of peptide molecular was assessed through multiple assays. The cytotoxicity of SA_2_-(PEG_2_)_2_-THA and SA_2_-PEG_2000_-CAR on MSCs was tested using a blade azurophilic assay at concentrations ranging from 5 to 100 μM. As illustrated in Figure 5C, cell viability remained close to 100% at the optimal peptide concentrations (20 μM for SA_2_-(PEG_2_)_2_-THA and 50 μM for SA_2_-PEG_2000_-CAR), indicating the absence of significant cytotoxicity.

To evaluate whether the hydrophobic insertion of SA_2_-PEG-Peptides disrupts cell membrane integrity, an LDH release assay was performed. As shown in Figure 5D, the LDH release rates for SA_2_-(PEG_2_)_2_-THA and SA_2_-PEG_2000_-CAR were below 2% after a 2-h incubation with MSCs at various concentrations. These results indicate that the hydrophobic insertion of the lipid-targeting peptides does not disrupt the membrane integrity of MSCs.

To evaluate the potential hemolytic effects of SA_2_-PEG-Peptides in blood circulation, hemolytic activity assays were conducted using erythrocytes derived from male Kunming mice. The erythrocytes were incubated for 1 h with varying concentrations of SA_2_-(PEG_2_)_2_-THA and SA_2_-PEG_2000_-CAR, while erythrocytes treated with Triton X-100 served as positive control. As illustrated in Figure 5E, both SA_2_-(PEG_2_)_2_-THA and SA_2_-PEG_2000_-CAR exhibited no significant hemolytic activity at a high concentration of 100 μM, with hemolysis rates remaining below 2%. These findings suggest that the SA_2_-PEG-Peptides possess favorable blood compatibility.

### 3.9 CAR-MSCs and PCAR-MSCs improve the homing and retention of MSCs in the lungs

**FIGURE 6:**
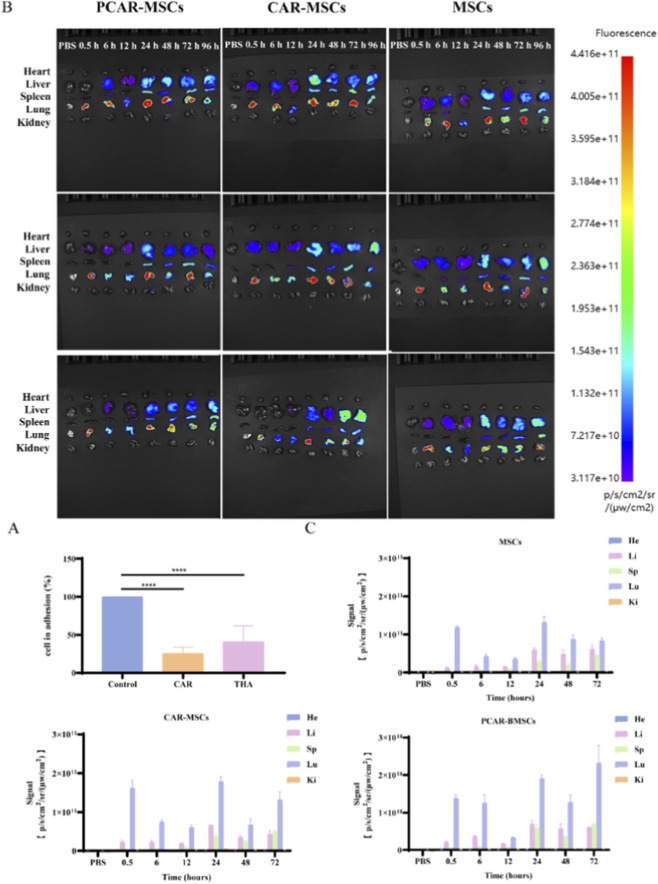
Cell adhesive assay and fluorescence distribution in various organs after tail vein injection of MSCs in mice. **(A)** Peptide-preincubated MSCs were seeded on collagen I (5 μg/cm^2^) monolayers and incubated for 30 min, adherent cells were then calculated (n = 4). **(B)** Fluorescence distribution maps of major organs (heart, liver, spleen, lung and kidney) observed by BioLive Imager of MSCs, CAR-MSCs and PCAR-MSCs at 0.5, 6, 12, 24, 48, 72 and 96 h after tail vein injection. Control group: PBS; MSCs group: simple MSCs not co-incubated with any drugs; CAR-MSCs group: 50 μM SA_2_-PEG_2000_-CAR incubated with MSCs for 30 min; PCAR-MSCs group: 50 μM SA_2_-PEG_2000_-CAR and 10 μM SA_2_-PEG_2000_ co-incubated for 30 min. **(C)** Quantitative analysis of ROI of fluorescence signals in each major organ at 0.5, 6, 12, 24, 48, 72, and 96 h after tail vein injection of MSCs, CAR-MSCs, and PCAR-MSCs, n = 3. He, heart; Li, liver; Sp, spleen; Lu, lung; Ki, kidney.

Modification of the cell surface by peptide drugs alters the adhesion capacity between cells and the extracellular matrix, which may result in intravenously infused MSCs becoming entrapped in the lungs and forming lethal microemboli (Gao et al., 2001; R. H. Lee et al., 2009). Therefore, assessing changes in cell adhesion capacity following incubation is of critical importance. As illustrated in Figure 6A, MSCs modified with SA_2_-PEG_2000_-CAR or SA_2_-(PEG_2_)_2_-THA were incubated at 37 °C for 30 min, followed by co-incubation with CI for an additional 30 min to evaluate their adhesion to CI. The results demonstrated that the adhesion capacity of MSCs modified with SA_2_-PEG_2000_-CAR was significantly reduced, thereby decreasing the probability of vascular embolism induced by the modified cells in this group.

The primary challenge in retaining MSCs following systemic administration arises from their phagocytosis and subsequent clearance by the reticuloendothelial system. Modification with PEG has been demonstrated to facilitate evasion of clearance by the monocyte-macrophage system. Prior to experimentation, screening of SA_2_-PEG_2000_-CAR was performed to evaluate its targeting efficiency and therapeutic potential *in vivo*.

A total volume of 200 μL, containing 1 × 10^6^ cells, resuspended in a 1:1 mixture of 1 mg/mL sodium heparin and PBS, was administered via tail vein injection in mice. The control group received injections of PBS alone. In the CAR-MSCs group, MSCs, pre-labeled with 30 μM DID for 2 h, were co-incubated with 50 μM SA_2_-PEG_2000_-CAR for 30 min prior to injection. For the PCAR-MSCs group, MSCs were co-incubated under identical conditions with both 50 μM SA_2_-PEG_2000_-CAR and 10 μM SA_2_-PEG_2000_ (DID-labeled). The MSCs group consisted solely of MSCs labeled with 30 μM DID.

As illustrated in Figures 6B,C, fluorescence signals following tail vein injections were predominantly localized in the lungs and liver from 0.5 to 96 h post-injection, with minimal fluorescence observed in the spleen and negligible signals observed in the heart and kidneys. These findings indicate that MSCs in the CAR-MSCs, PCAR-MSCs, and control groups were primarily distributed within the lungs. These findings indicate that MSCs in the CAR-MSCs, PCAR-MSCs, and control groups were primarily distributed within the lungs.

**FIGURE 7:**
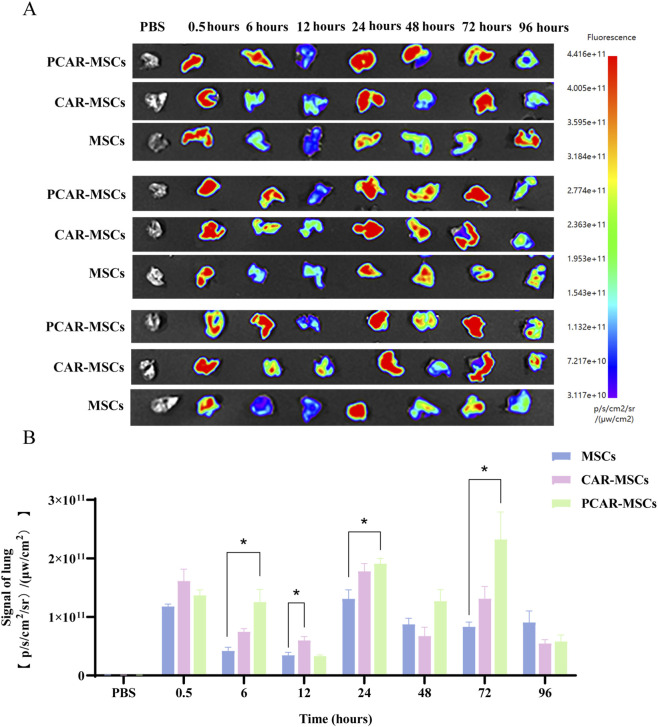
Fluorescence signal distribution maps and quantitative analysis of lung tissues after tail vein injection of MSCs in mice. **(A)** Fluorescence signals in the lungs of MSCs, CAR-MSCs, and PCAR-MSCs at 0.5, 6, 12, 24, 48, 72, and 96 h after tail vein injection were observed by Bioinvivo Imaging. Control group: PBS; MSCs group: simple MSCs not co-incubated with any drugs; CAR-MSCs group: 50 μM SA_2_-PEG_2000_-CAR incubated with MSCs for 30 min; PCAR-MSCs group: 50 μM SA_2_-PEG_2000_-CAR and 10 μM SA_2_-PEG_2000_ co-incubated for 30 min. **(B)** Fluorescence signals in the lungs of MSCs, CAR-MSCs, and PCAR-MSCs at 0.5, 6, 12, 24, 48, 72, and 96 h after tail vein injection ROI quantitative analysis, n = 3. *p < 0.05, indicating that this experimental group exhibited statistical differences compared to the MSCs group.

To further evaluate lung homing and retention, fluorescence intensity within lung tissues was analyzed (Figure 7). Compared to the MSCs group, both CAR-MSCs and PCAR-MSCs exhibited significantly enhanced lung fluorescence signals up to 72 h post-injection, with PCAR-MSCs showing the most pronounced increase. These results propose that SA_2_-PEG_2000_-CAR modification improves MSCs homing to the lungs, while co-modification with SA_2_-PEG_2000_ further enhances retention. This dual modification strategy may augment the therapeutic efficacy of MSC-based treatments for IPF.

### 3.10 PCAR-MSCs enhance IPF treatment-efficiency

Compared to unmodified MSCs, PCAR-MSCs exhibited enhanced lung homing and retention capabilities. To investigate whether CAR-MSCs and PCAR-MSCs could enhance therapeutic efficacy in an IPF mouse model, MSC-based treatments were administered. IPF was experimentally induced in mice through the tracheal administration of BLM. Seven days following induction, the mice were administered tail vein injections of PBS, MSCs, CAR-MSCs, or PCAR-MSCs at a dosage of 1 × 10^6^ cells per injection, delivered once weekly for a total of three doses.

**FIGURE 8:**
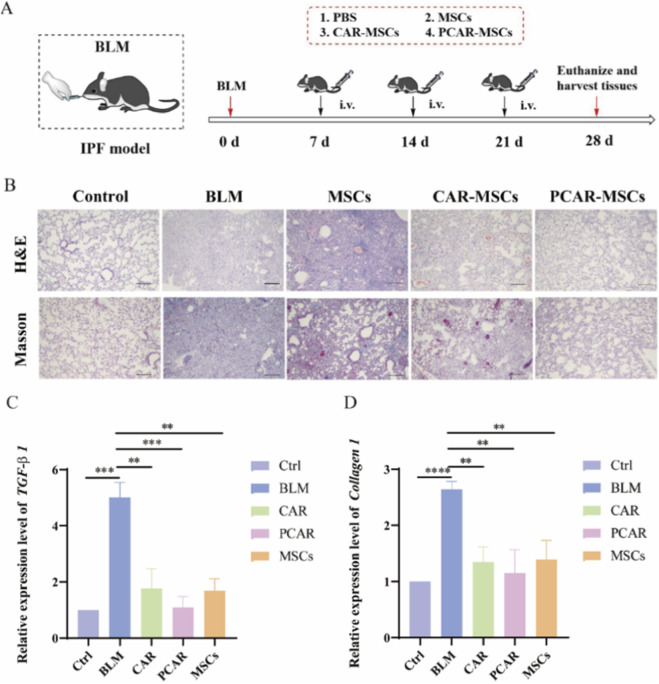
*In vivo* therapeutic capacity of IPF for lung-targeted MSCs. **(A)** Schematic diagram of IPF modeling and MSCs administration. **(B)** BLM and H&E and Masson staining of lung tissues of MSCs, CAR-MSCs, and PCAR-MSCs treatment groups. Analysis of morphological changes in lung tissues of mice, n = 3. Scale bar: 100 μm. Control group: PBS; BLM group: bleomycin; MSCs group: simple MSCs not co-incubated with any drugs; CAR-MSCs group: 50 μM SA_2_-PEG_2000_-CAR incubated with MSCs for 30 min; PCAR-MSCs group: 50 μM SA_2_-PEG_2000_-CAR and 10 μM SA_2_-PEG_2000_ co-incubated for 30 min. **(C)** Relative expression levels of *TGF-β 1*. **(D)** Relative expression levels of *Collagen 1*. The transcription level of the ctrl was defined as 1.0. Experiments were performed in biological triplicates, and error bars indicate the standard deviations. Asterisks indicate statistically significant differences compared to the WT: **P < 0.01; ***P < 0.001; ****P ≤ 0.0001.

After 4 weeks, lung tissues were collected and analyzed using H&E and Masson staining (Figure 8B). In H&E-stained tissues, the BLM group exhibited characteristic IPF pathology, including alveolar wall thickening, infiltration of inflammatory cells, and disruption of alveolar architecture. In contrast, lung tissues from the CAR-MSCs and PCAR-MSCs groups demonstrated marked histological improvement. Notably, the PCAR-MSCs group exhibited tissue morphology comparable to that of the healthy control group, characterized by reduced inflammatory infiltration, diminished fibrosis, and preserved alveolar walls.

Masson staining further confirmed that both CAR-MSCs and PCAR-MSCs significantly reduced fibrotic areas, with PCAR-MSCs exhibiting the most pronounced improvement. These results indicate that CAR modification effectively enhances the homing of MSCs to lung tissues, whereas PEG modification increases the retention of MSCs at the disease site. This combined strategy enhances the therapeutic efficacy of MSCs in treating IPF by prolonging their functional presence within the lungs.

As illustrated in Figures 7C,D, the expression levels of IPF-related genes, specifically *TGF-β1* and *collagen 1*, were reduced in the group treated with MSCs compared to the BLM-treated group, approaching those observed in the control (Ctrl) group. These results suggest that stem cell treatment exerts a reparative effect on pulmonary fibrosis. Notably, the expression levels in the CAR and PCAR groups were closer to those in the Ctrl group, with the PCAR group demonstrating the lowest expression levels.

In conclusion, the co-modification of MSCs with SA_2_-PEG_2000_-CAR and SA_2_-PEG_2000_ represents an effective strategy to enhance MSC homing and retention within the lungs, thereby improving therapeutic outcomes for IPF.

## 4 Discussion

MSCs therapy has increasingly demonstrated considerable potential in the treatment of various diseases and clinical applications, presenting a promising alternative for conditions lacking effective pharmacological interventions (Le Blanc et al., 2004). Nevertheless, numerous studies and clinical trials have indicated that transplanted MSCs exhibit inadequate homing to pathological sites and possess limited retention capacity *in vivo* (Fang et al., 2025; Fu et al., 2023; Yu et al., 2023). These limitations result in rapid clearance by the reticuloendothelial system, thereby reducing therapeutic efficacy (Goldman et al., 2021). Cell surface modification, which entails the attachment of functional molecules to the cell membrane, can regulate critical cellular processes including differentiation, proliferation, adhesion, and interactions with neighboring cells and biomolecules (Fang et al., 2025; H. Han et al., 2025; M.-M. Han et al., 2023; J. Liao et al., 2025; Sagaradze et al., 2020; H. Wang et al., 2020; Wu et al., 2025). Consequently, cell surface modification of MSCs may provide an effective strategy to enhance their homing ability and retention at disease sites, ultimately improving the therapeutic outcomes of MSC-based treatments (Lemos and Duffield, 2018; El Agha et al., 2017; Lin et al., 2011; Rabelink and Little, 2013; Toonkel et al., 2013).

**FIGURE 9:**
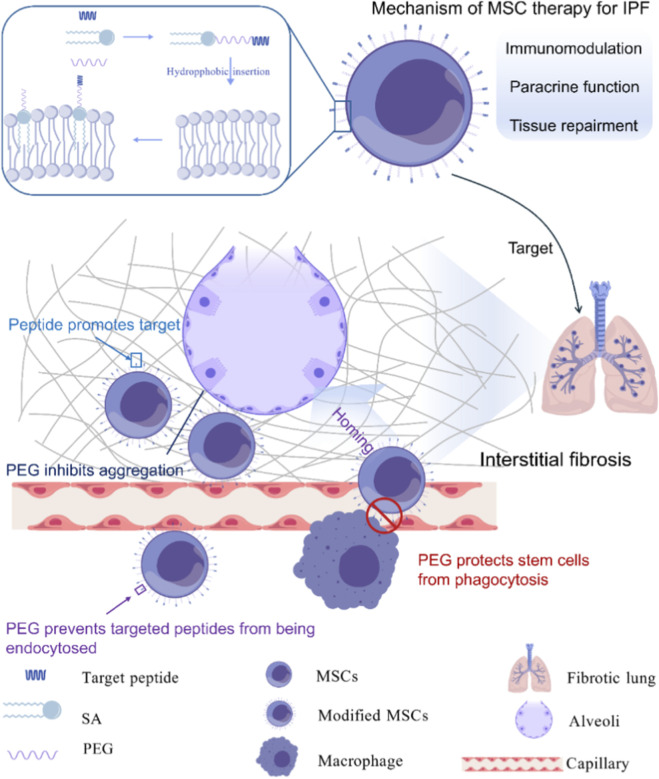
Lung-targeting peptides and pegylated surface modifications enhance homing and retention of MSCs in lung tissue: a comprehensive overview.

Our experiments demonstrated that Lipid-PEG-Peptides effectively modified the surfaces of MSCs via hydrophobic insertion, thereby facilitating the efficient incorporation of lung-targeting peptides into the MSC membrane and thereby enhancing the cells’ homing ability to lung tissue. PEG component imparts hydrophilicity and modulates the internalization of SA_2_-PEG-Peptides, resulting in increased peptide retention on the cell membrane. Additionally, the long PEG chains provide sufficient spatial flexibility, enabling lung-targeting peptides lung-targeting peptides to fully exert their targeting functions. Although systemically administered MSCs accumulate within the alveolar capillary network, they are typically rapidly cleared by macrophage-mediated phagocytosis. Surface modification of MSCs with SA_2_-PEG forms a protective PEGylated shell that evades clearance by the monocyte-macrophage system. Specifically, SA_2_-PEG_2000_ modification inhibits macrophage-mediated phagocytosis and prolongs MSC retention in lung tissues. The combined modification strategy, employing both SA_2_-PEG-Peptides and SA_2_-PEG_2000_, significantly enhances MSC homing and retention in lung tissues following systemic administration (Figure 9, comprehensive overview is drawn by GDP - Generic Diagramming Platform).

This study aimed to improve the homing and retention of MSCs in lung tissues following systemic administration via cell surface modification using lung-targeted peptides. Two primary cell surface engineering strategies were employed: metabolic glycan engineering combined with copper-free orthogonal click chemistry, and lipid-PEG hydrophobic modification. Microscopic imaging analyses confirmed the successful conjugation of 5-FAM fluorescently labeled DBCO and SA_2_ to the MSC surface, thereby validating the applicability of both strategies to MSCs.

Further experiments investigated the concentration dependence of Ac_4_ManNAz and DBCO incubation using microscopic imaging and flow cytometry. To assess the cellular retention of the first strategy, flow cytometry was used to measure the retention time on MSCs surfaces. Results indicated that the metabolic glycan engineering strategy, when combined with copper-free orthogonal click chemistry, resulted in a retention time of less than 24 h. Due to its inefficiency in MSCs-targeting and the complexity of the procedure, focus shifted to the second strategy, Lipid -PEG hydrophobic modification. Subsequent investigations revealed that SA_2_-PEG_2000_-CAR, applied in the Lipid-PEG modification, conferred enhanced retention on the MSC surface.

The vascular homing peptide CAR exhibits penetrative capacity penetrate cells and tissues, including granulation tissue at wound sites and remodeled pulmonary vascular walls in rats with pulmonary arterial hypertension (PAH) (Urakami et al., 2011). CAR induces syndecan-4-dependent activation of the small GTPase ARF6 via the guanine nucleotide exchange factor celladon-2, and promoting keratinocyte migration mediated by syndecan-4, ARF6, and cytohesin-2. Thereby, systemically administered CAR peptides selectively target pulmonary tissue (Jost et al., 2001; Maldonado et al., 2023).

The incorporation of hydrophilic PEG chains was identified as a critical factor in inhibiting cellular internalization, while the length of the PEG chains modulated the efficiency of cellular modification. We synthesized SA_2_-PEG-Peptides and their analogs with varying PEG chain lengths, coupling positions, and quantities. Comparative analyses of these analogues demonstrated that PEG chain length, lipid coupling position, and the number of lipid moieties significantly affect cellular internalization. Targeting peptides with N-terminal lipid attachment and PEG doping typically exhibited lower cellular internalization.

To achieve optimal modification efficiency, the effects of incubation concentration and time were assessed for SA_2_-(PEG_2_)_2_-THA and SA_2_-PEG_2000_-CAR. Flow cytometry and microscopic imaging results indicated that these couplers exhibited varying degrees of internalization based on concentration and incubation time. Further examination of cytotoxicity, membrane integrity, and hemolytic activity identified the optimal conditions: 50 μM SA_2_-(PEG_2_)_2_-THA for 15 min and 50 μM SA_2_-PEG_2000_-CAR for 30 min. Modification efficiencies were 69.35% and 75.15%, respectively. Consequently, SA_2_-PEG_2000_-CAR was selected for further experiments.

*In vivo* targeting efficacy was evaluated using bioimaging, where exhibited a marked increase in lung tissue fluorescence at 72 h post-injection. Numerous studies have leveraged the hydrophilicity of PEG to self-assemble it into nanoparticles for drug delivery (M.-M. Han et al., 2023; He et al., 2024; Nguyen et al., 2024; Nguyen et al., 2023; Yu et al., 2023), or to prepare liposomes by incorporating it as a membrane component (Fang et al., 2025; H. Han et al., 2025). Given this, we propose directly modifying it onto the surface of stem cell membranes (Takayama et al., 2023). The long PEG chains reduce endocytosis of surface-modified peptides due to membrane fluidity, forming a protective shell on the stem cell surface. Treating MSCs as the delivered drug, targeted peptides enable precise delivery to the lesion site. Leveraging the stem cells’ self-repair capabilities facilitates tissue repair at the diseased site. In the IPF model, the group treated with PCAR-MSCs demonstrated superior therapeutic outcomes, characterized by diminished lung structural damage, preservation of alveolar walls, and reduced fibrosis. These findings suggest that CAR peptide modification effectively enhances MSC homing to lung tissues, thereby decreasing the required transplantation dose while increasing therapeutic efficacy. Furthermore, PEGylation of MSCs further improved their retention at disease sites, resulting in sustained therapeutic benefits over time.

In conclusion, surface modification with SA_2_-PEG_2000_-CAR and SA_2_-PEG_2000_ significantly improved MSCs lung targeting and retention. Lung-targeted PCAR-MSCs demonstrated improved therapeutic efficacy in the treatment of IPF, highlighting the potential of the SA_2_-PEG-peptide strategy for broader applications in cell modification and disease therapy.

## 5 Conclusion

This study develops a co-modification strategy using SA_2_-PEG_2000_-CAR and SA_2_-PEG_2000_ SA_2_-PEG_2000_-CAR and SA_2_-PEG_2000_ to enhance the therapeutic potential of MSCs for IPF. The optimized PEG configuration improved cell surface modification, leading to significantly increased lung homing and retention of MSCs after systemic administration. As a result, MSCs modified with PCAR exhibited significantly enhanced effectiveness in addressing IPF, presenting a promising and versatile framework for cutting-edge stem cell therapies.

## Data Availability

The original contributions presented in the study are included in the article/Supplementary Material, further inquiries can be directed to the corresponding author.

## References

[B1] Chan J. L. Tang K. C. Patel A. P. Bonilla L. M. Pierobon N. Ponzio N. M. (2006). Antigen-presenting property of mesenchymal stem cells occurs during a narrow window at low levels of interferon-γ. Blood 107 (12), 4817–4824. 10.1182/blood-2006-01-0057 16493000 PMC1895812

[B2] Chen Y. Wang T. Liang F. Han J. Lou Z. Yu Y. (2024). Nicotinamide phosphoribosyltransferase prompts bleomycin-induced pulmonary fibrosis by driving macrophage M2 polarization in mice. Theranostics 14 (7), 2794–2815. 10.7150/thno.94482 38773984 PMC11103509

[B3] El Agha E. Kramann R. Schneider R. K. Li X. Seeger W. Humphreys B. D. (2017). Mesenchymal stem cells in fibrotic disease. Cell Stem Cell 21 (2), 166–177. 10.1016/j.stem.2017.07.011 28777943

[B4] Fang Y. Zhang C. Han M. Wang Y. Zhou T. Xing L. (2025). Engineered MSCs break endothelial-myofibroblast crosstalk in pulmonary fibrosis: reconstructing the vascular niche. Adv. Mater. 37, e2414601. 10.1002/adma.202414601 40018848

[B5] Fu Z. Zhang Y. Geng X. Chi K. Liu C. Song C. (2023). Optimization strategies of mesenchymal stem cell-based therapy for acute kidney injury. Stem Cell Res. Ther. 14 (1), 116. 10.1186/s13287-023-03351-2 37122024 PMC10150535

[B6] Galipeau J. Sensebe L. (2018). Mesenchymal stromal cells: clinical challenges and therapeutic opportunities. Cell Stem Cell 22 (6), 824–833. 10.1016/j.stem.2018.05.004 29859173 PMC6434696

[B7] Gao J. Dennis J. E. Muzic R. F. Lundberg M. Caplan A. I. (2001). The dynamic *in vivo* distribution of bone marrow-derived mesenchymal stem cells after infusion. Cells Tissues Organs 169 (1), 12–20. 10.1159/000047856 11340257

[B8] Glass D. S. Grossfeld D. Renna H. A. Agarwala P. Spiegler P. DeLeon J. (2022). Idiopathic pulmonary fibrosis: current and future treatment. Clin. Respir. J. 16 (2), 84–96. 10.1111/crj.13466 35001525 PMC9060042

[B9] Goldman D. L. Aldrich M. L. Hagmann S. H. F. Bamford A. Camacho-Gonzalez A. Lapadula G. (2021). Compassionate use of remdesivir in children with severe COVID-19. Pediatrics 147 (5), e2020047803. 10.1542/peds.2020-047803 33883243

[B10] Gunatilaka A. Zhang S. Tan W. S. D. G Stewart A. (2023). Anti-fibrotic strategies and pulmonary fibrosis. Adv. Pharmacol. 98, 179–224. 10.1016/bs.apha.2023.04.002 37524487

[B11] Gutmann M. Bechold J. Seibel J. Meinel L. Luehmann T. (2019). Metabolic glycoengineering of cell-derived matrices and cell surfaces: a combination of key principles and step-by-step procedures. ACS Biomater. Sci. Eng. 5 (1), 215–233. 10.1021/acsbiomaterials.8b00865 33405877

[B12] Han M.-M. He X.-Y. Tang L. Qi L. Yang M.-Y. Wang Y. (2023). Nanoengineered mesenchymal stem cell therapy for pulmonary fibrosis in young and aged mice. Sci. Adv. 9 (29), eadg5358. 10.1126/sciadv.adg5358 37467328 PMC10355834

[B13] Han H. Chen B.-T. Liu Y. Qi L. Xing L. Wang H. (2025). Engineered stem cell booster breaks pathological barriers to treat chronic pancreatitis. Adv. Mater 37 (14), e2416261. 10.1002/adma.202416261 40012418

[B14] He X.-Y. Han M.-M. Zhao Y.-C. Tang L. Wang Y. Xing L. (2024). Surface-engineered mesenchymal stem cell for refractory asthma therapy: reversing airway remodeling. J. Control. Release 376, 972–984. 10.1016/j.jconrel.2024.10.056 39476873

[B15] Honig B. Shapiro L. (2020). Adhesion protein structure, molecular affinities, and principles of cell-cell recognition. Cell 181 (3), 520–535. 10.1016/j.cell.2020.04.010 32359436 PMC7233459

[B16] Hulugalla K. Shofolawe-Bakare O. Toragall V. B. Mohammad S. A. Mayatt R. Hand K. (2024). Glycopolymeric nanoparticles enrich less immunogenic protein coronas, reduce mononuclear phagocyte clearance, and improve tumor delivery compared to pegylated nanoparticles. ACS Nano 18 (44), 30540–30560. 10.1021/acsnano.4c08922 39436672 PMC12045476

[B17] Hwang B. W. Kim S. J. Park K. M. Kim H. Yeom J. Yang J.-A. (2015). Genetically engineered mesenchymal stem cell therapy using self-assembling supramolecular hydrogels. J. Control. Release 220, 119–129. 10.1016/j.jconrel.2015.10.034 26485045

[B18] Jarvinen T. A. H. Ruoslahti E. (2007). Molecular changes in the vasculature of injured tissues. Am. J. Pathol. 171 (2), 702–711. 10.2353/ajpath.2007.061251 17600129 PMC1934529

[B19] Ji X. Wang L. Zhong Y. Xu Q. Yan J. Pan D. (2024). Impact of mesenchymal stem cell size and adhesion modulation on *in vivo* distribution: insights from quantitative PET imaging. Stem Cell Res. Ther. 15 (1), 456. 10.1186/s13287-024-04078-4 39609885 PMC11606219

[B20] Jingxuan L. Zewen W. Li Z. Yang L. Yazhen S. Xueyan G. (2023). The heterogeneity of mesenchymal stem cells: an important issue to be addressed in cell therapy. Stem Cell Res. Ther. 14 (1), 381. 10.1186/s13287-023-03587-y 38124129 PMC10734083

[B21] Jost P. J. Harbottle R. P. Knight A. Miller A. D. Coutelle C. Schneider H. (2001). A novel peptide, THALWHT, for the targeting of human airway epithelia. FEBS Lett. 489 (2-3), 263–269. 10.1016/S0014-5793(00)02236-5 11165262

[B22] Kapoor D. U. Gandhi S. M. Swarn S. Lal B. Prajapati B. G. Khondee S. (2025). Polymeric nanoparticles for targeted lung cancer treatment: review and perspectives. Cells-Basel 17 (9), 1091. 10.3390/pharmaceutics17091091 41012430 PMC12472848

[B23] Kim S. Kim K. (2022). Lipid-mediated *ex vivo* cell surface engineering for augmented cellular functionalities. Mat. Sci. Eng. C-Mater 140, 213059. 10.1016/j.bioadv.2022.213059 35961186

[B24] Kim D. H. Kim H. C. Im K. Baek I. J. Choi Y. J. Lee H. (2025). Inhibition of AXL ameliorates pulmonary fibrosis via attenuation of M2 macrophage polarisation. Eur. Respiratory J. 65 (6), 2400615. 10.1183/13993003.00615-2024 39788632 PMC12138030

[B25] Kufleitner M. Haiber L. M. Wittmann V. (2023). Metabolic glycoengineering - exploring glycosylation with bioorthogonal chemistry. Chem. Soc. Rev. 52 (2), 510–535. 10.1039/d2cs00764a 36537135

[B26] Lamb Y. N. (2021). Nintedanib: a review. Drugs 81 (5), 575–586. 10.1007/s40265-021-01487-0 33765296 PMC8163683

[B27] Le Blanc K. Rasmusson I. Sundberg B. Götherström C. Hassan M. Uzunel M. (2004). Treatment of severe acute graft-versus-host disease with third party haploidentical mesenchymal stem cells. Lancet 363 (9419), 1439–1441. 10.1016/S0140-6736(04)16104-7 15121408

[B28] Lee R. H. Pulin A. A. Seo M. J. Kota D. J. Ylostalo J. Larson B. L. (2009). Intravenous hMSCs improve myocardial infarction in mice because cells embolized in lung are activated to secrete the anti-inflammatory protein TSG-6. Cell Stem Cell 5 (1), 54–63. 10.1016/j.stem.2009.05.003 19570514 PMC4154377

[B29] Lee D. Y. Cha B.-H. Jung M. Kim A. S. Bull D. A. Won Y.-W. (2018). Cell surface engineering and application in cell delivery to heart diseases. J. Biol. Eng. 12, 28. 10.1186/s13036-018-0123-6 30524502 PMC6278044

[B30] Lemos D. R. Duffield J. S. (2018). Tissue-resident mesenchymal stromal cells: implications for tissue-specific antifibrotic therapies. Sci. Transl. Med. 10 (426), eaan5174. 10.1126/scitranslmed.aan5174 29386358

[B31] Li M. Shang X. Lou H. Wang Z. Xiang S. Qiu Y. (2024). Active anchoring stimuli-responsive nano-craft to relieve pulmonary vasoconstriction by targeting smooth muscle cell for hypoxic pulmonary hypertension treatment. Adv. Healthc. Mater. 13 (15), e2400113. 10.1002/adhm.202400113 38412500

[B32] Liao N. S. Zhang D. Wu M. Yang H. H. Liu X. L. Song J. B. (2021). Enhancing therapeutic effects and *in vivo* tracking of adipose tissue-derived mesenchymal stem cells for liver injury using bioorthogonal click chemistry. NanoScale 13 (3), 1813–1822. 10.1039/d0nr07272a 33433536

[B33] Liao J. Zhu Z. Zou J. Liu S. Luo X. Bao W. (2025). Macrophage membrane-biomimetic multi-layered nanoparticles targeting synovial angiogenesis for osteoarthritis therapy. Adv. Healthc. Mater. 14 (2), e2401985. 10.1002/adhm.202401985 39402771

[B34] Lim S. Yoon H. Y. Park S.-J. Song S. Shim M. K. Yang S. (2021). Predicting *in vivo* therapeutic efficacy of bioorthogonally labeled endothelial progenitor cells in hind limb ischemia models via non-invasive fluorescence molecular tomography. Biomaterials 266, 120472. 10.1016/j.biomaterials.2020.120472 33120201

[B35] Lin H. Xu R. Zhang Z. Chen L. Shi M. Wang F. S. (2011). Implications of the immunoregulatory functions of mesenchymal stem cells in the treatment of human liver diseases. Cell. Mol. Immunol. 8 (1), 19–22. 10.1038/cmi.2010.57 21200380 PMC4002992

[B36] Liu Q. Bi Y. Song S. Zhu K. Qiao X. Wang H. (2023). Exosomal miR-17-5p from human embryonic stem cells prevents pulmonary fibrosis by targeting thrombospondin-2. Stem Cell Res. Ther. 14 (1), 234. 10.1186/s13287-023-03449-7 37667335 PMC10478444

[B37] Liu K. Meng X. Liu Z. Tang M. Lv Z. Huang X. (2024). Tracing the origin of alveolar stem cells in lung repair and regeneration. Cell 187 (10), 2428–2445.e20. 10.1016/j.cell.2024.1003.1010 38579712

[B38] Lu Y. Chen T. Lin H. Chen Y. Lin Y. Le D. (2025). Small extracellular vesicles engineered using click chemistry to express chimeric antigen receptors show enhanced efficacy in acute liver failure. J. Extracell. Vesicles 14 (2), e70044. 10.1002/jev2.70044 39901768 PMC11791321

[B39] Luchini A. Vitiello G. (2021). Mimicking the mammalian plasma membrane: an overview of lipid membrane models for biophysical studies. Biomimetics 6 (1), 3. 10.3390/biomimetics6010003 33396534 PMC7838988

[B40] Maher T. M. (2024). Interstitial lung disease: a review. Jama-Jam Med. Assoc. 331 (19), 1655–1665. 10.1001/jama.2024.3669 38648021

[B41] Maldonado H. Savage B. D. Barker H. R. May U. Vahatupa M. Badiani R. K. (2023). Systemically administered wound-homing peptide accelerates wound healing by modulating syndecan-4 function. Nat. Commun. 14 (1), 8069. 10.1038/s41467-023-43848-1 38057316 PMC10700342

[B42] Melis S. Trompet D. Chagin A. S. Maes C. (2025). Skeletal stem and progenitor cells in bone physiology, ageing and disease. Nat. Rev. Endocrinol. 21 (3), 135–153. 10.1038/s41574-024-01039-y 39379711

[B43] Németh K. Leelahavanichkul A. Yuen P. S. T. Mayer B. Parmelee A. Doi K. (2009). Bone marrow stromal cells attenuate sepsis via prostaglandin E(2)-dependent reprogramming of host macrophages to increase their interleukin-10 production. Nat. Med. 15 (1), 42–49. 10.1038/nm.1905 19098906 PMC2706487

[B44] Nguyen T. T. Phuong M. T. Shrestha M. Park J. Le Minh P. Kim J. O. (2023). Scalable and uniform fabrication of dexamethasone-eluting depot-engineered stem cell spheroids as a microtissue construct to target bone regeneration. ACS Appl. Mater Inter 15 (22), 26373–26384. 10.1021/acsami.3c03102 37219569

[B45] Nguyen T. T. Kil Y.-S. Sung J.-H. Youn Y. S. Jeong J. H. Lee J. H. (2024). Fabrication of stem cell heterospheroids with sustained-release chitosan and poly(lactic-co-glycolic acid) microspheres to guide cell fate toward chondrogenic differentiation. Int. J. Biol. Macromol. 263, 130356. 10.1016/j.ijbiomac.2024.130356 38395283

[B46] Oga T. Matsuoka T. Yao C. Nonomura K. Kitaoka S. Sakata D. (2009). Prostaglandin F(2alpha) receptor signaling facilitates bleomycin-induced pulmonary fibrosis independently of transforming growth factor-beta. Nat. Med. 15 (12), 1426–1430. 10.1038/nm.2066 19966781

[B47] Ouji-Sageshima N. Hiyama A. Kumamoto M. Kitabatake M. Hara A. Furukawa R. (2024). Adipose-derived mesenchymal stem cells (ADSCs) have anti-fibrotic effects on lung fibroblasts from idiopathic pulmonary fibrosis (IPF) patients. Cells-Basel 13 (24), 2050. 10.3390/cells13242050 39768142 PMC11674916

[B48] Pei X. L. Zheng F. X. Li Y. Lin Z. J. Han X. Feng Y. (2022). Niclosamide ethanolamine salt alleviates idiopathic pulmonary fibrosis by modulating the PI3K-mTORC1 pathway. Cells-Basel 11 (3), 346. 10.3390/cells11030346 35159160 PMC8834116

[B49] Rabelink T. J. Little M. H. (2013). Stromal cells in tissue homeostasis: balancing regeneration and fibrosis. Nat. Rev. Nephrol. 9 (12), 747–753. 10.1038/nrneph.2013.152 23938596

[B50] Sagaradze G. D. Basalova N. A. Efimenko A. Y. Tkachuk V. A. (2020). Mesenchymal stromal cells as critical contributors to tissue regeneration. Front. Cell Dev. Biol. 8, 576176. 10.3389/fcell.2020.576176 33102483 PMC7546871

[B51] Takayama Y. Kusamori K. Katsurada Y. Obana S. Itakura S. Nishikawa M. (2023). Efficient delivery of mesenchymal stem/stromal cells to injured liver by surface PEGylation. Stem Cell Res. Ther. 14 (1), 216. 10.1186/s13287-023-03446-w 37608303 PMC10464485

[B52] Tian Z. Zenan Y. Jianyu W. Duanqing P. Xin D. Chang H. (2021). Challenges and advances in clinical applications of mesenchymal stromal cells. J. Hematol. Oncol. 14 (1), 24. 10.1186/s13045-021-01037-x 33579329 PMC7880217

[B53] Toba M. Alzoubi A. O'Neill K. Abe K. Urakami T. Komatsu M. (2014). A novel vascular homing peptide strategy to selectively enhance pulmonary drug efficacy in pulmonary arterial hypertension. Am. J. Pathol. 184 (2), 369–375. 10.1016/j.ajpath.2013.10.008 24401613 PMC3906494

[B54] Toonkel R. L. Hare J. M. Matthay M. A. Glassberg M. K. (2013). Mesenchymal stem cells and idiopathic pulmonary fibrosis potential for clinical testing. Am. J. Resp. Crit. Care 188 (2), 133–140. 10.1164/rccm.201207-1204PP 23306542

[B55] Urakami T. Jarvinen T. A. H. Toba M. Sawada J. Ambalavanan N. Mann D. (2011). Peptide-directed highly selective targeting of pulmonary arterial hypertension. Am. J. Pathol. 178 (6), 2489–2495. 10.1016/j.ajpath.2011.02.032 21549345 PMC3123986

[B56] Wang L.-T. Ting C.-H. Yen M.-L. Liu K.-J. Sytwu H.-K. Wu K. K. (2016). Human mesenchymal stem cells (MSCs) for treatment towards immune- and inflammation-mediated diseases: review of current clinical trials. J. Biomed. Sci. 23, 76. 10.1186/s12929-016-0289-5 27809910 PMC5095977

[B57] Wang H. Shang Y. Chen X. Wang Z. Zhu D. Liu Y. (2020). Delivery of MSCs with a hybrid β-sheet peptide hydrogel consisting IGF-1C domain and D-form peptide for acute kidney injury therapy. Int. J. Nanomed. 15, 4311–4324. 10.2147/ijn.S254635 32606679 PMC7306577

[B58] Wu C. X. Huang Z. W. Chen J. M. Li N. Cai Y. Chen J. L. (2025). Efficiently directing differentiation and homing of mesenchymal stem cells to boost cartilage repair in osteoarthritis via a nanoparticle and peptide dual-engineering strategy. Biomaterials 312, 122720. 10.1016/j.biomaterials.2024.122720 39084098

[B59] Yu S. Yu S. Liu H. Liao N. Liu X. (2023). Enhancing mesenchymal stem cell survival and homing capability to improve cell engraftment efficacy for liver diseases. Stem Cell Res. Ther. 14 (1), 235. 10.1186/s13287-023-03476-4 37667383 PMC10478247

[B60] Zhao X. Kwan J. Y. Y. Yip K. Liu P. P. Liu F. F. (2020). Targeting metabolic dysregulation for fibrosis therapy. Nat. Rev. Drug Discovery 19 (1), 57–75. 10.1038/s41573-019-0040-5 31548636

[B61] Zhong Y. Qin X. Wang Y. Qu K. Luo L. Zhang K. (2021). “Plug and Play” functionalized erythrocyte nanoplatform for target atherosclerosis management. ACS Appl. Mater. Inter. 13 (29), 33862–33873. 10.1021/acsami.1c07821 34256560

