# Supplementary material for: Targeted peptide modification of mesenchymal stem cells enhances their therapeutic efficacy in the treatment of idiopathic pulmonary fibrosis
Source: Front Cell Dev Biol. 2025 Dec 18;13:1710750. doi: 10.3389/fcell.2025.1710750 (PMC12756429; doi:10.3389/fcell.2025.1710750)
Supplement: Supplementary file 1 [file DataSheet1.pdf]

Table S1 Name and sequence of peptide

**Name**	**Sequence**
THA	H-THALWHT-OH
FAM-THA	FAM-Acp-K(DBCO)-THALWHT-OH
SA_2-(PEG_2)$_2$-THA	SA_2-K-(PEG_2)$_2$-K-THALWHT-OH
SA_2-(PEG_2)$_2$-K(FAM)-THA	SA_2-K-(PEG_2)$_2$-K(FAM)-THALWHT-OH
SA_2-PEG_{2000}-THA	SA_2-K-PEG_{2000}-K-THALWHT-OH
SA_2-PEG_{2000}-K(FAM)-THA	SA_2-K-PEG_{2000}-K(FAM)-THALWHT-OH
FAM-CAR	FAM-Acp-CARSKNKDC-OH
SA_2-(PEG_2)$_2$-CAR	SA_2-K-(PEG_2)$_2$-K-CARSKNKDC-OH
SA_2-(PEG_2)$_2$-K(FAM)-CAR	SA_2-K-(PEG_2)$_2$-K(FAM)-CARSKNKDC-OH
SA_2-PEG_{2000}-CAR	SA_2-K-PEG_{2000}-K-CARSKNKDC-OH
SA_2-PEG_{2000}-K(FAM)-CAR	SA_2-K-PEG_{2000}-K(FAM)-CARSKNKDC-OH

Table S2 Retention time, purity and ESI-MS results of RP-HPLC analysis of targeted peptides

**Name**	**RT (min)**	**Purity (%)**	**Exact mass**	**ESI-MS ($M+H^+$)**
THA	11.575	96.531	864.42	865.4360
FAM-THA	16.032	95.570	1750.7456	1751.7162
SA_2-(PEG_2)$_2$-THA	34.177	94.329	1943.28	1944.2774
SA_2-(PEG_2)$_2$-K(FAM)-THA	33.128	97.229	2301.33	2302.3120
SA_2-PEG_{2000}-THA	31.945	98.641	3647.31	4154.2514
SA_2-PEG_{2000}-K(FAM)-THA	33.732	99.278	4005.36	4007.3254
FAM-CAR	13.550	98.628	1381.50	1382.5204
SA_2-(PEG_2)$_2$-CAR	22.298	96.715	2102.32	2103.3086
SA_2-(PEG_2)$_2$-K(FAM)-CAR	22.227	94.286	2460.37	2461.3373
SA_2-PEG_{2000}-CAR	32.870	98.429	3806.35	3807.3109
SA_2-PEG_{2000}-K(FAM)-CAR	33.542	93.970	4164.39	4170.0707

The structures, HPLC analysis and MS spectra of the peptides in the table are shown in Fig S10-20.

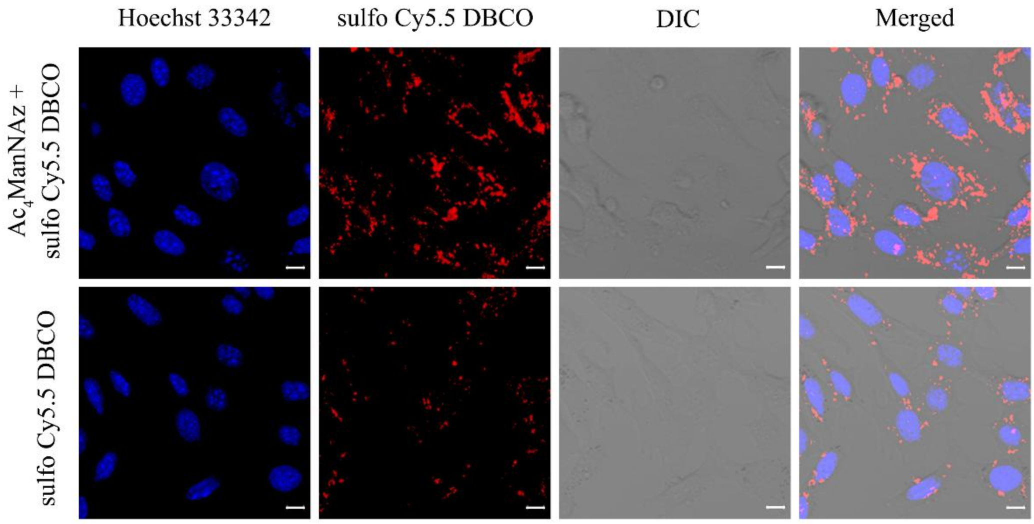

Fig. S1 Laser confocal fluorescence imaging of stem cell modifications based on metabolic glycoengineering and bioorthogonal click chemistry. 20 μM Ac4ManNAz was incubated with MSCs for 72 h and then washed, and 20 μM sulfo-Cy5.5-DBCO was added to react with azide groups generated on the cell membranes in a copper-free click chemistry reaction with an incubation time of 1 h. Cells not pretreated with Ac4ManNAz served as controls. Red: Sulfo Cy5.5-DBCO; blue: Hoechst 33342. scale bar: 20 μm.

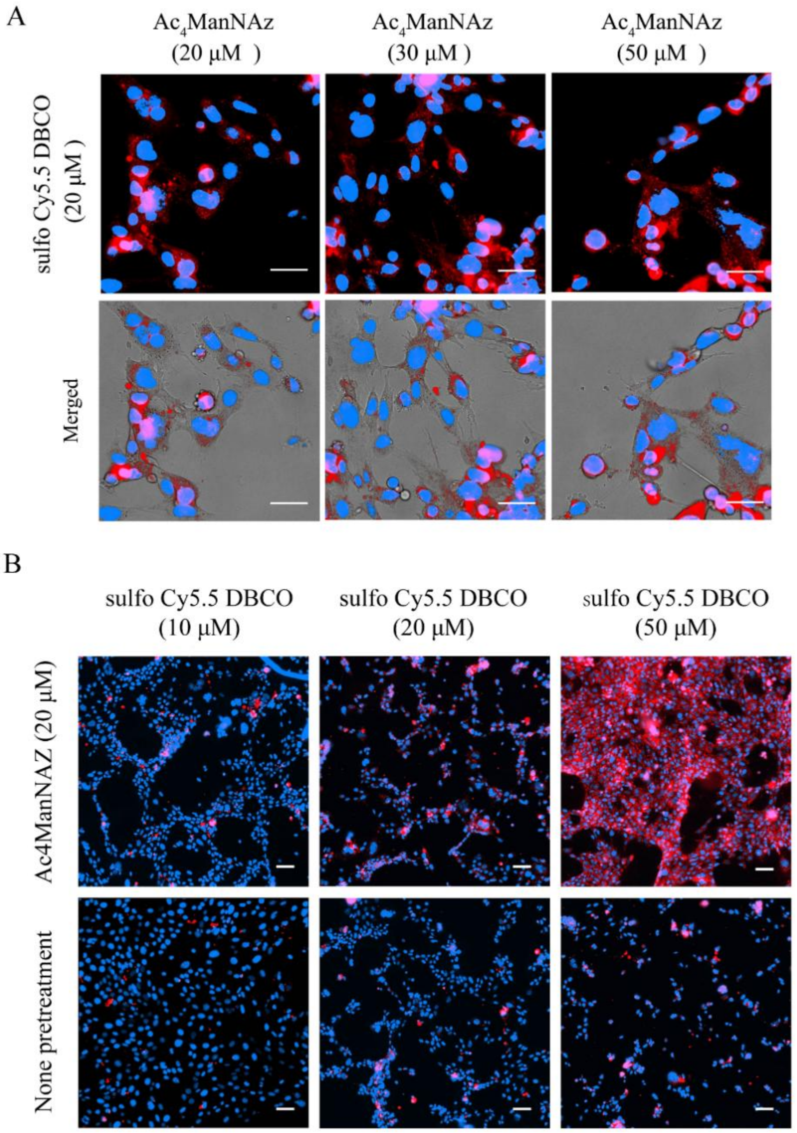

Fig. S2 Condition optimization (A) Optimization of Ac4ManNAz conditions. Fluorescence images of Ac4ManNAz incubated with MSCs at different concentrations (20, 30, 50 μM) and 20 μM sulfo-Cy5.5-DBCO (incubated for 1 h) bioorthogonal click chemistry were observed by High Intensity Cell Imager. Red: Sulfo-Cy5.5-DBCO; blue: Hoechst 33342. scale bar: 20 μm. (B) Optimization of sulfo-Cy5.5-DBCO bioorthogonal click chemistry conditions. 20 μM Ac4ManNAz was incubated with MSCs for 3 days after incubation for 1 h at different sulfo-Cy5.5-DBCO concentrations (20, 30, 50 μM) were incubated for 1 h. Fluorescence images of bioorthogonal click chemistry MSCs. Fluorescence changes were observed under a high content cell imager. Red: Sulfo-Cy5.5-DBCO; blue: Hoechst 33342. scale bar: 50 μm.

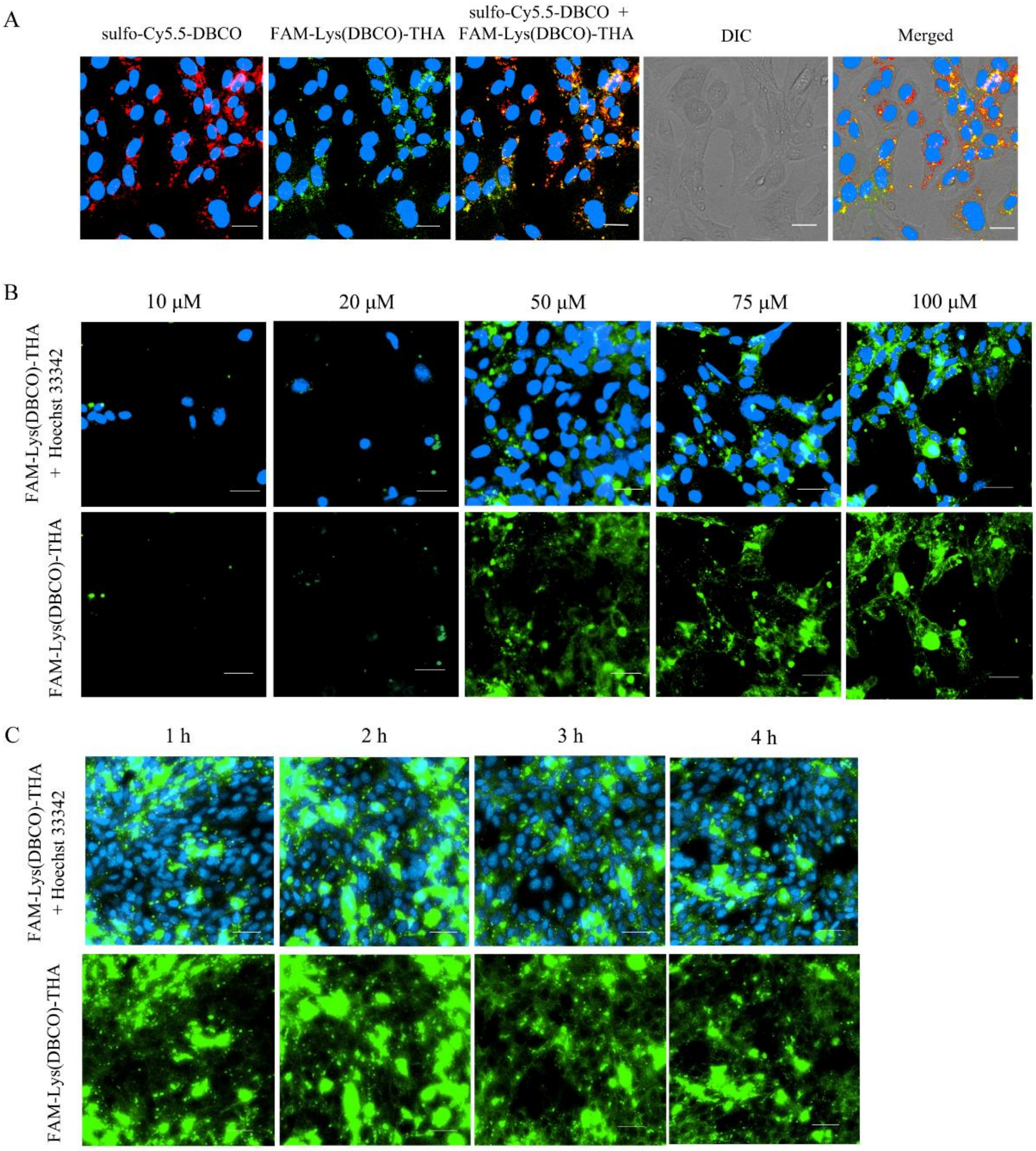

Fig. S3 FAM-Lys(DBCO)-THA cell membrane co-localization and optimization of conditions. (A) Bioorthogonal click chemistry confirmation of Ac4ManNAz and FAM-Lys(DBCO)-THA in MSCs. after MSCs were incubated with 20 μM Ac4ManNAz for 3 days, 25 μM DBCO-Cy5.5 was co-incubated with 25 μM FAM-Lys(DBCO)-THA in MSCs for 1 h. Under the high endo-cellular imager fluorescence co-localization was observed. Red: Sulfo Cy5.5-DBCO; green: FAM-Lys(DBCO)-THA; blue: Hoechst 33342. scale bar: 20 μm. (B) MSCs were incubated with 20 μM Ac4ManNAzfor 3 days after the generation of azide groups on the cell surface. MSCs were incubated with different concentrations (20, 30, 50, 75, 100 μM) of FAM-Lys(DBCO)-THA for 1 h. Fluorescence co-localization was observed under a high content cell imager. (C) 20 μM Ac4ManNAz was incubated with MSCs for 3 days followed by incubation with 50 μM of FAM-Lys(DBCO)-THA for 1, 2, 3, and 4 h. Observed under a high content cell imager. Red: Sulfo Cy5.5-DBCO; green: FAM-Lys(DBCO)-THA; blue: Hoechst 33342. scale bar: 20 μm.

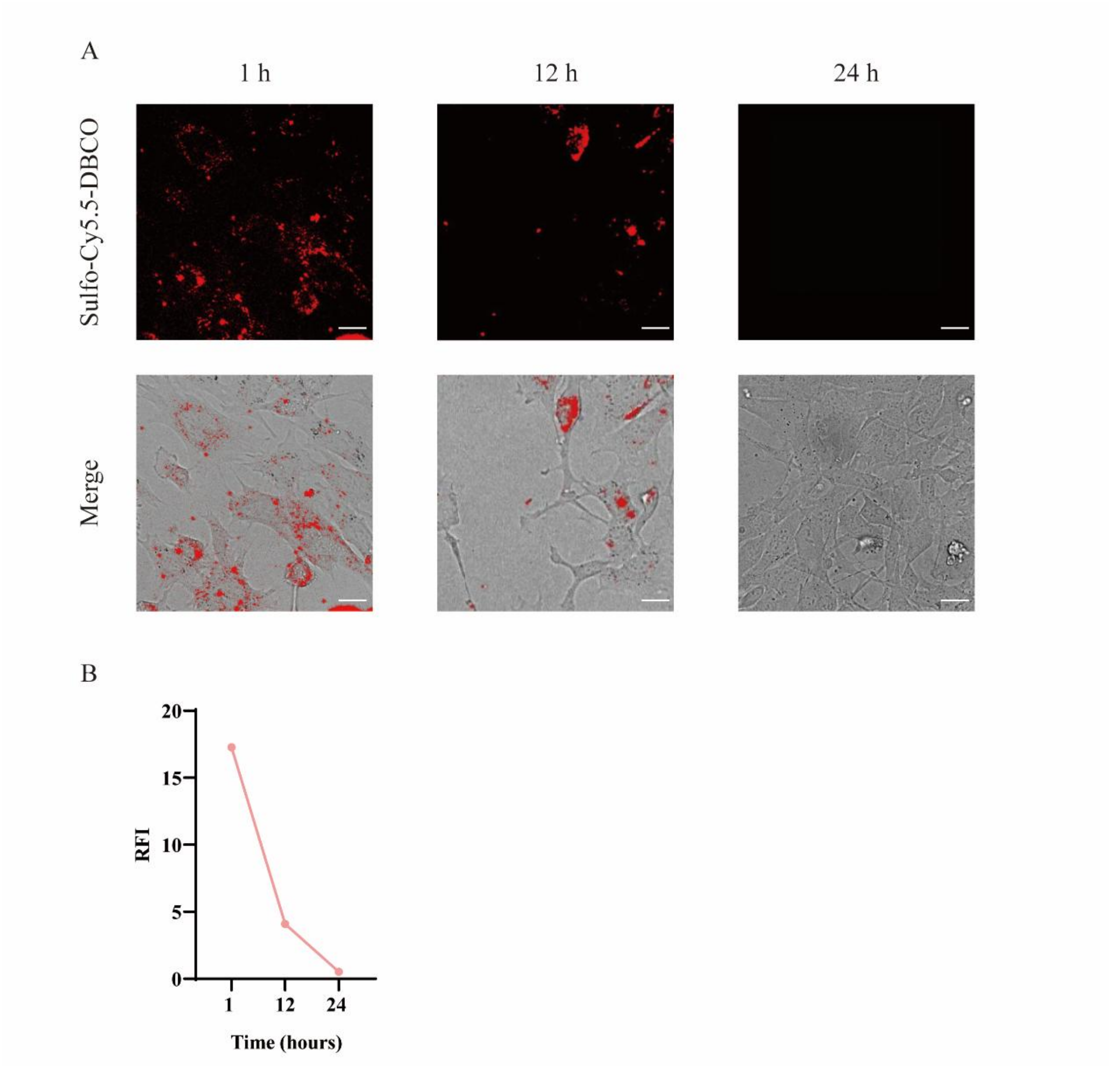

Fig. S4 MGE modifies fluorescence retention time. (A) MSCs were incubated with Ac4ManNAz (20 μM) for three days, via sulfo-Cy5.5-DBCO (50 μM) for 1 h. High-content cell imager was used to observe the retention of intrafluorescence on MSCs for 24 h. The fluorescence retention time was analyzed by Image J. The fluorescence retention time was analyzed by Image J. The fluorescence retention time was analyzed by Image J. Red: sulfo-Cy5.5-DBCO. (B) Image J analysis of the average fluorescence intensity in (A). Scale bar: 20 μm.

THA: H-THALWHT-OH

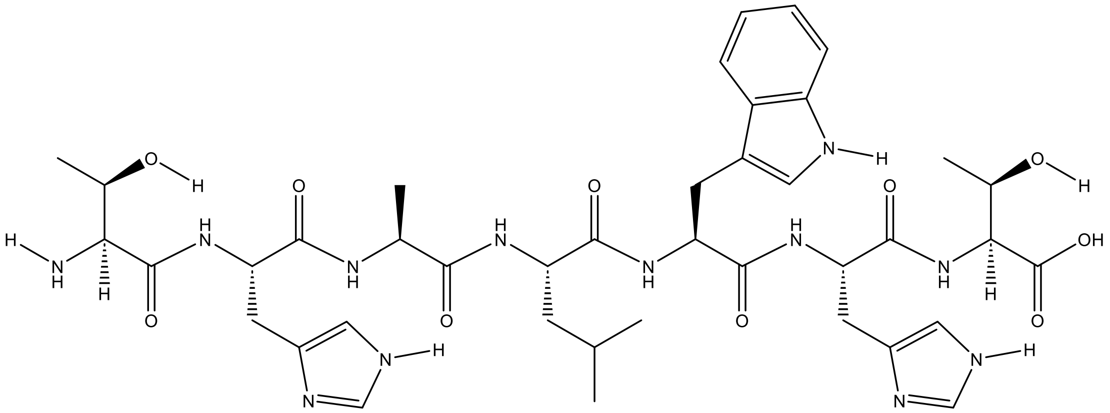

Chemical Formula: $C_{40}H_{56}N_{12}O_{10}$

Exact Mass: 864.4242, $M+H^{+}$=865.4360

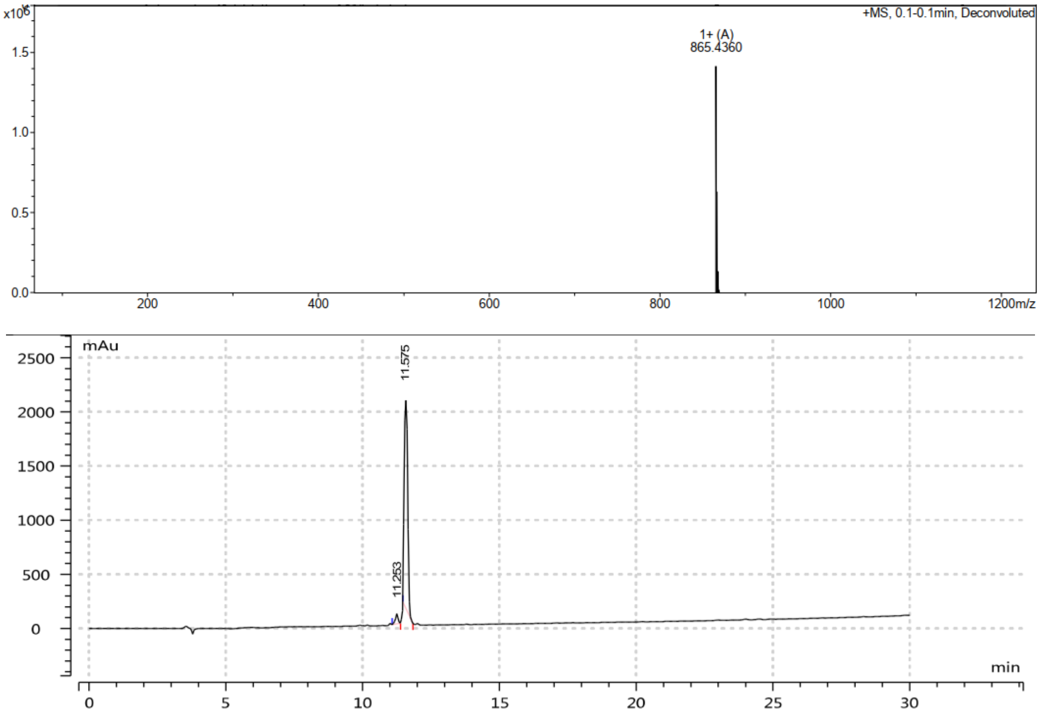

Fig. S10 Structure, HPLC analysis and MS spectrum of THA

FAM-THA: FAM-Acp-K(DBCO)-THALWHT-OH

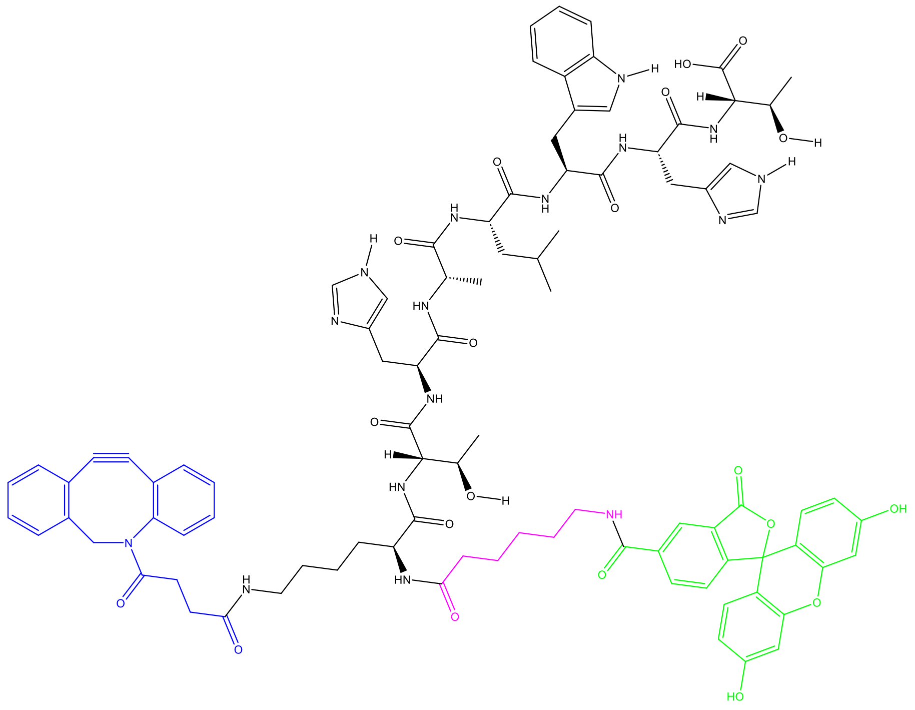

Chemical Formula: $C_{92}H_{102}N_{16}O_{20}$

Exact Mass: 1750.7456, M+H^{+}=1751.7162

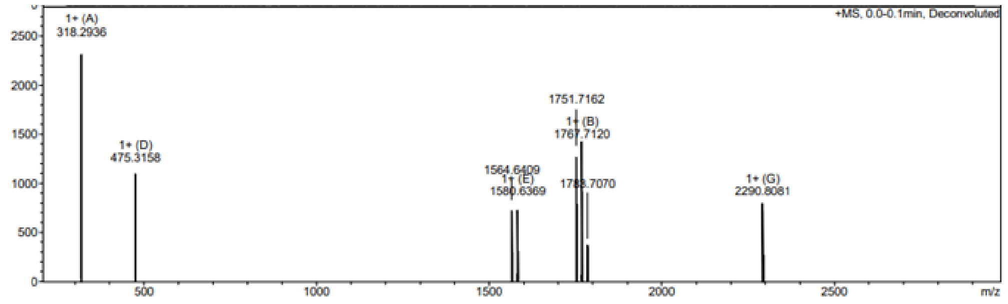

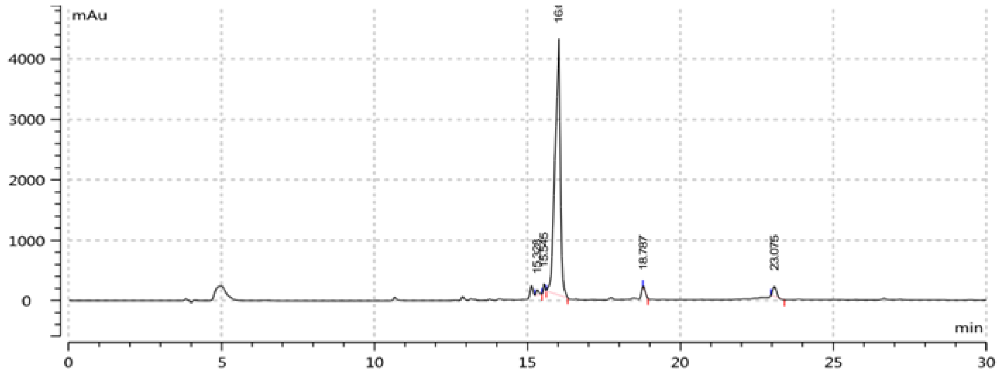

Fig. S11 Structure, HPLC analysis and MS spectrum of FAM-THA

SA$_2$-(PEG$_2$)$_2$-THA: SA$_2$-K-(PEG$_2$)$_2$-K-THALWHT-OH

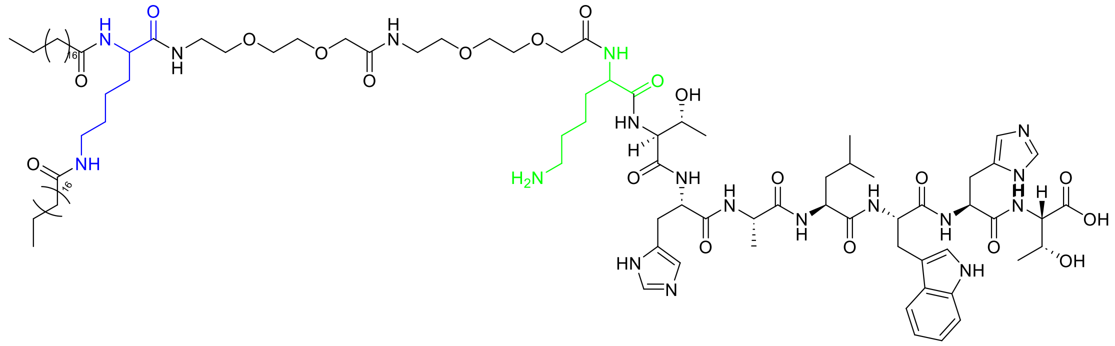

Chemical Formula: $C_{100}H_{170}N_{18}O_{20}$

Exact Mass: 1943.28, M+H$^+$=1944.2774

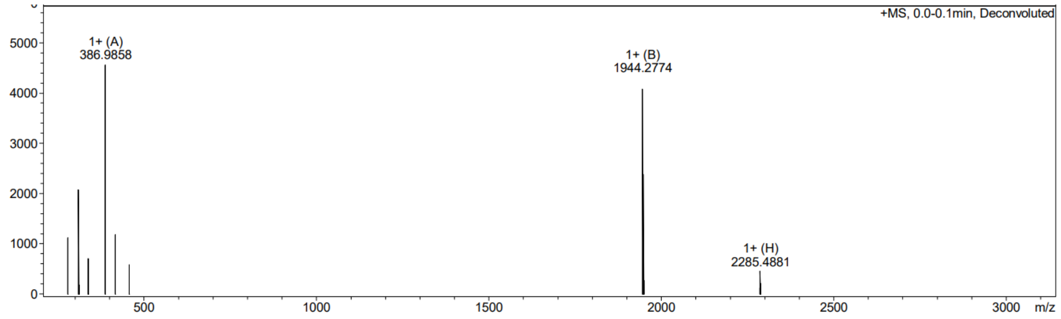

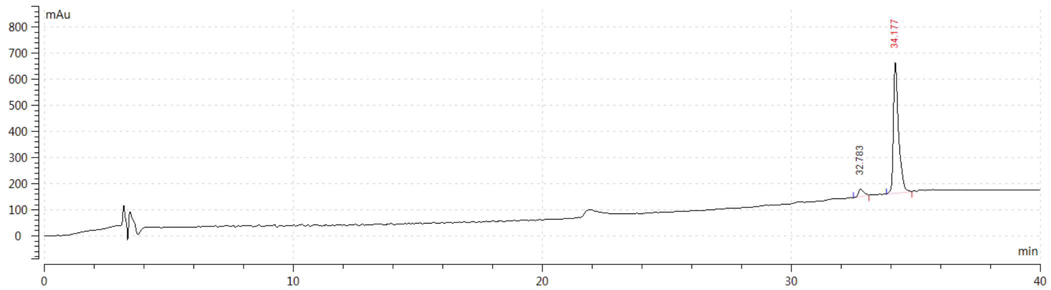

Fig. S12 Structure, HPLC analysis and MS spectrum of SA$_2$-(PEG$_2$)$_2$-THA

SA$_2$-(PEG$_2$)$_2$-K(FAM)-THA: SA$_2$-K-(PEG$_2$)$_2$-K(FAM)-THALWHT-OH

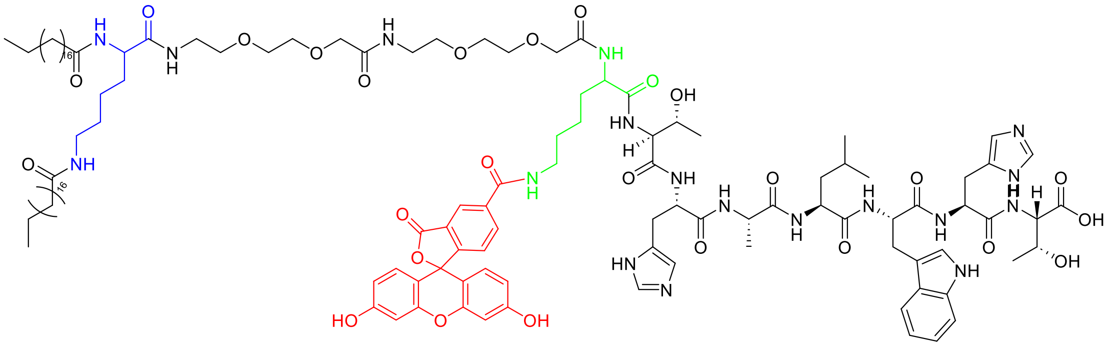

Chemical Formula: $C_{121}H_{180}N_{18}O_{26}$

Exact Mass: 2301.33, M+H$^+$=2302.3120

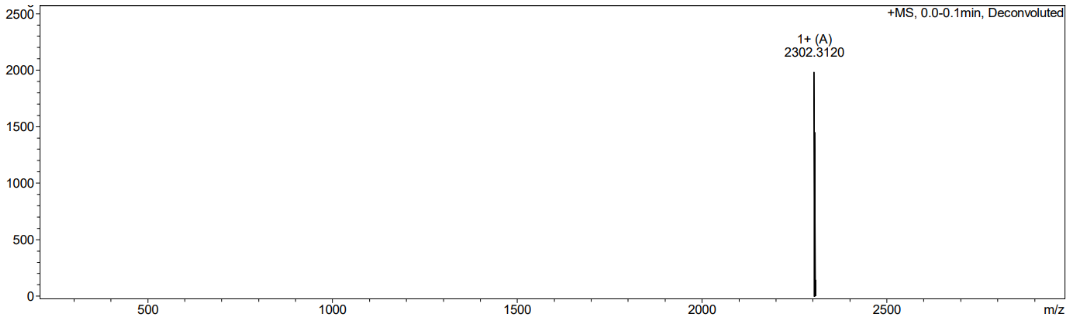

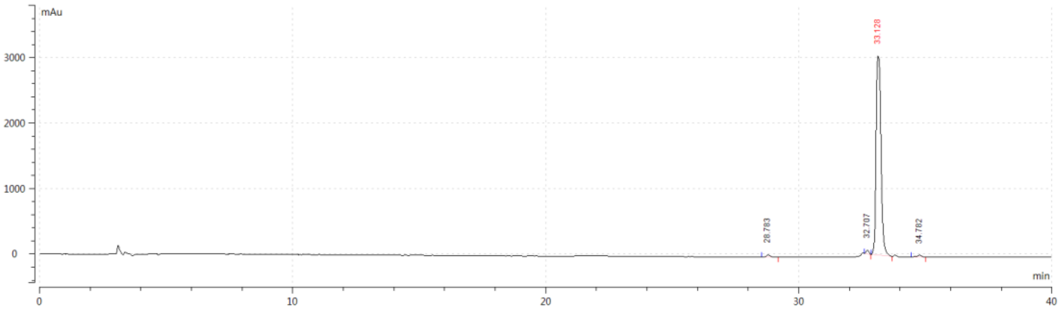

Fig. S13 Structure, HPLC analysis and MS spectrum of SA$_2$-(PEG$_2$)$_2$-K(FAM)-THA

SA$_2$-PEG$_{2000}$-THA: SA$_2$-K-PEG$_{2000}$-K-THALWHT-OH

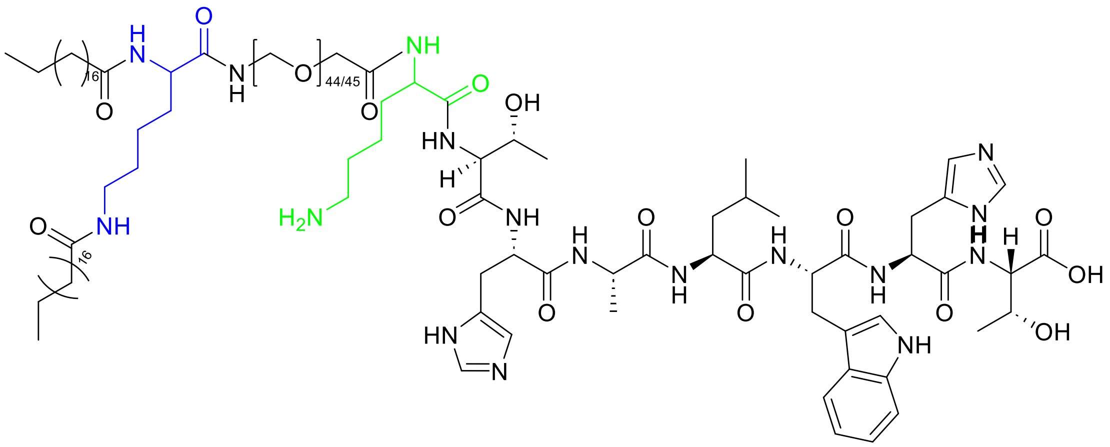

Chemical Formula: $C_{178}H_{327}N_{17}O_{59}$

Exact Mass: 3647.31, M+H$^+$=3648

Due to the nature of the PEG2000 product, which is a series of products differing by 44. Here 4154 is 11.5 times more than 3648, which may be due to detector instability, but this result is still correct.

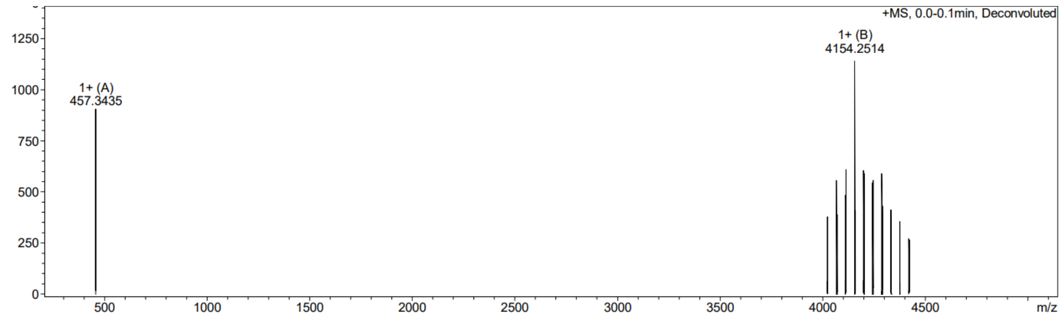

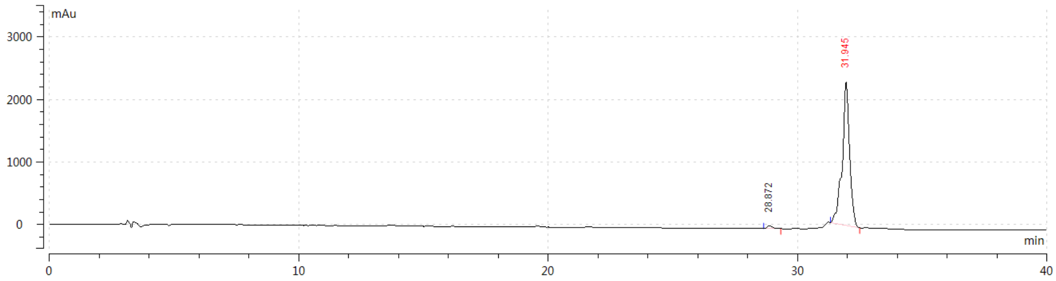

Fig. S14 Structure, HPLC analysis and MS spectrum of SA$_2$-PEG$_{2000}$-THA

SA$_2$-PEG$_{2000}$-K(FAM)-THA: SA$_2$-K-PEG$_{2000}$-K(FAM)-THALWHT-OH

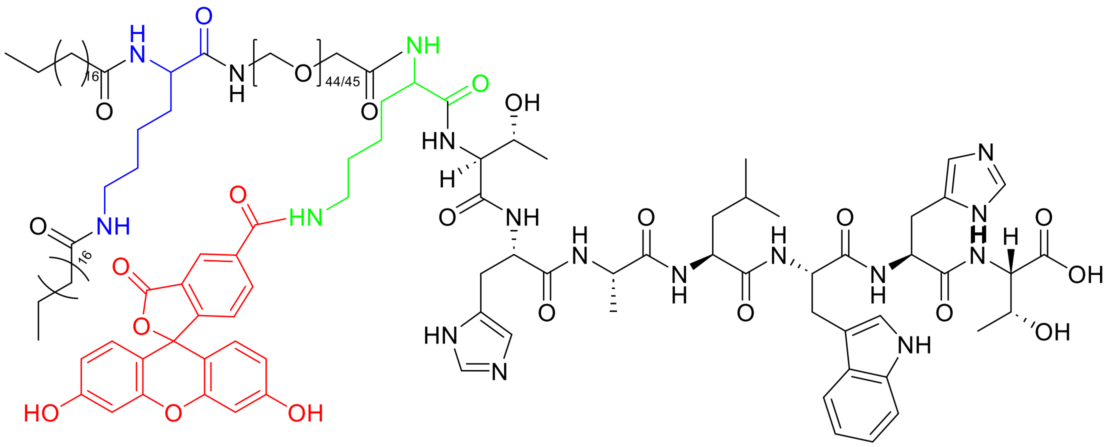

Chemical Formula: $C_{199}H_{337}N_{17}O_{65}$

Exact Mass: 4005.36, M+H$^+$=4006

Here 4007 is only one molecular weight larger than 4006, which may be due to detector instability, but this result is still correct.

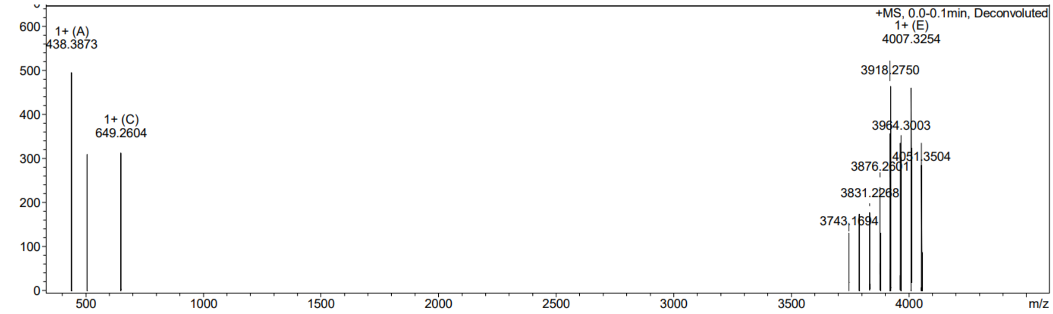

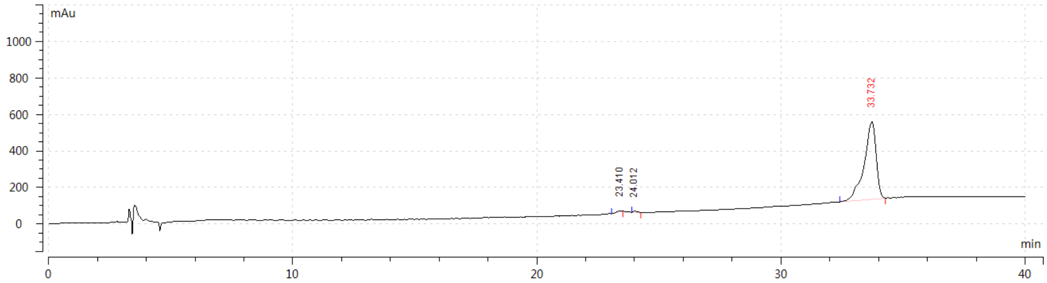

Fig. S15 Structure, HPLC analysis and MS spectrum of SA$_2$-PEG$_{2000}$-K(FAM)-THA

FAM-CAR: FAM-Acp-CARSKNKDC-OH

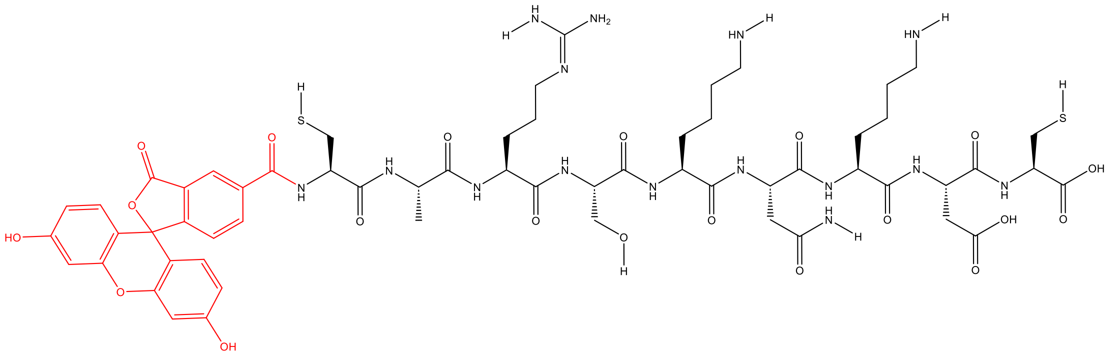

Chemical Formula: $C_{59}H_{79}N_{15}O_{20}S_2$

Exact Mass: 1381.5067, $M+H^+$=1382.5204

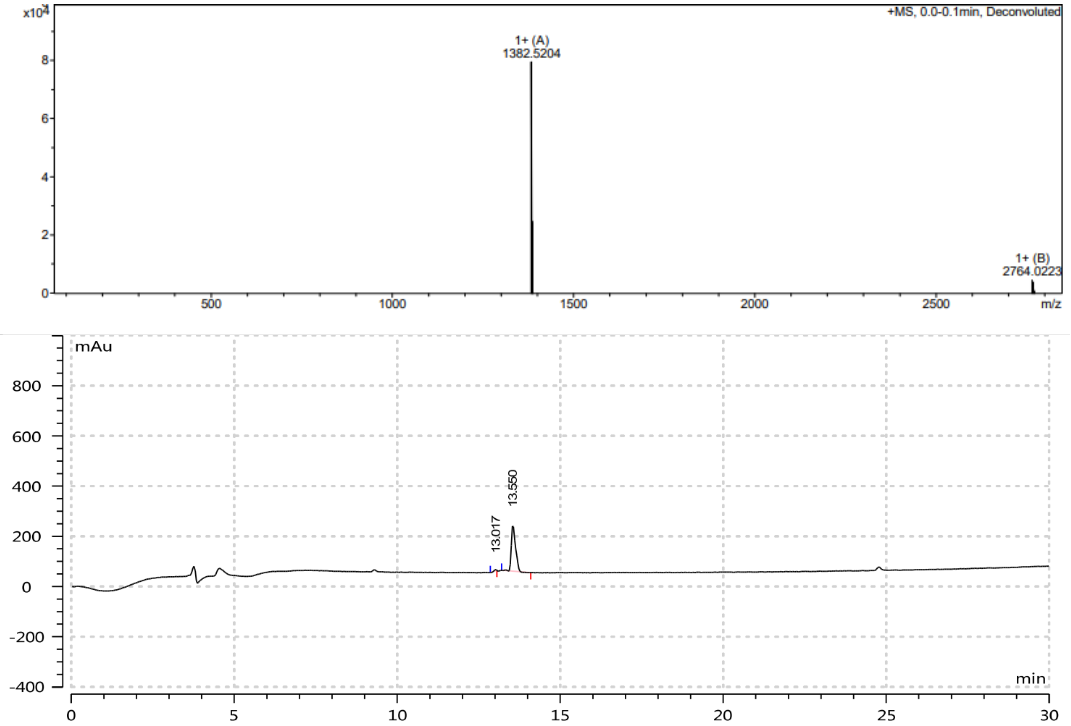

Fig. S16 Structure, HPLC analysis and MS spectrum of FAM-CAR

SA$_2$-(PEG$_2$)$_2$-CAR: SA$_2$-K-(PEG$_2$)$_2$-K-CARSKNKDC-OH

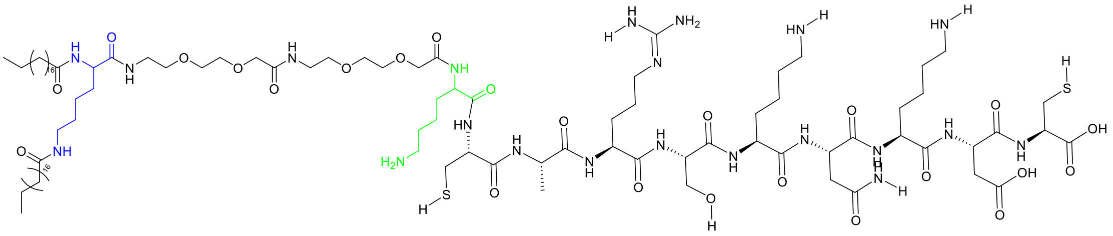

Chemical Formula: $C_{98}H_{183}N_{21}O_{24}S_2$

Exact Mass: 2102.32, M+H$^+$=2103.3086

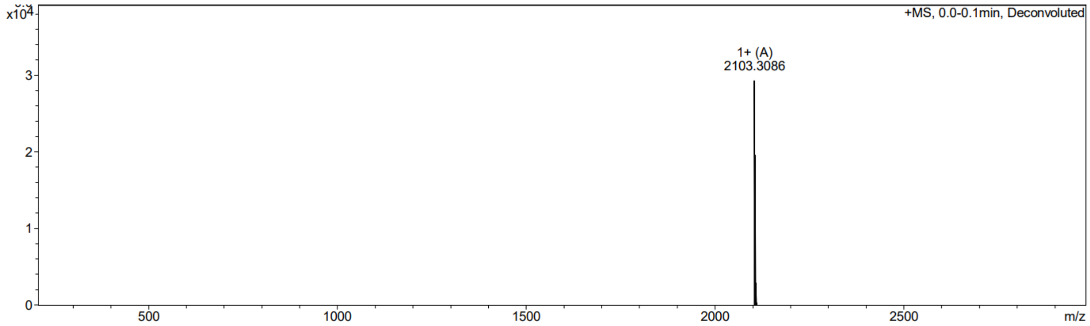

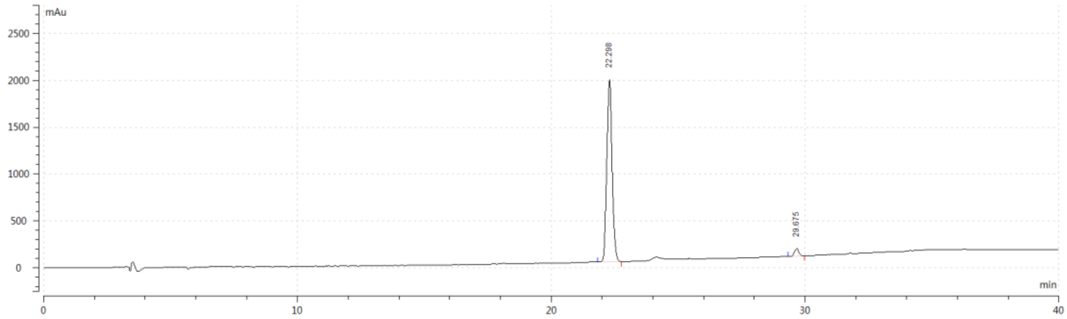

Fig. S17 Structure, HPLC analysis and MS spectrum of SA$_2$-(PEG$_2$)$_2$-CAR

SA$_2$-(PEG$_2$)$_2$-K(FAM)-CAR: SA$_2$-K-(PEG$_2$)$_2$-K(FAM)-CARSKNKDC-OH

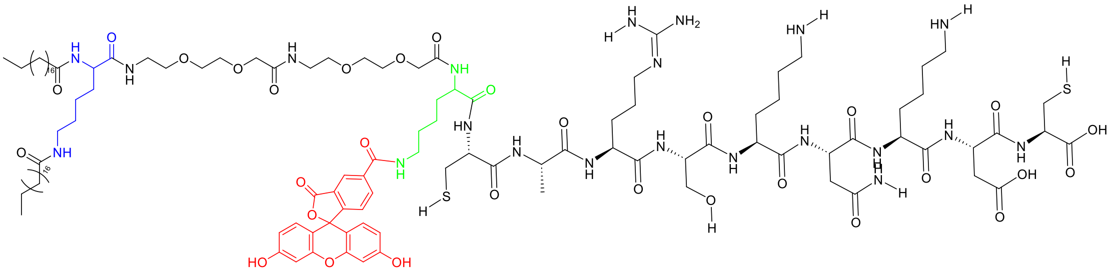

Chemical Formula: $C_{119}H_{193}N_{21}O_{30}S_2$

Exact Mass: 2460.37, M+H$^+$=2461.3373

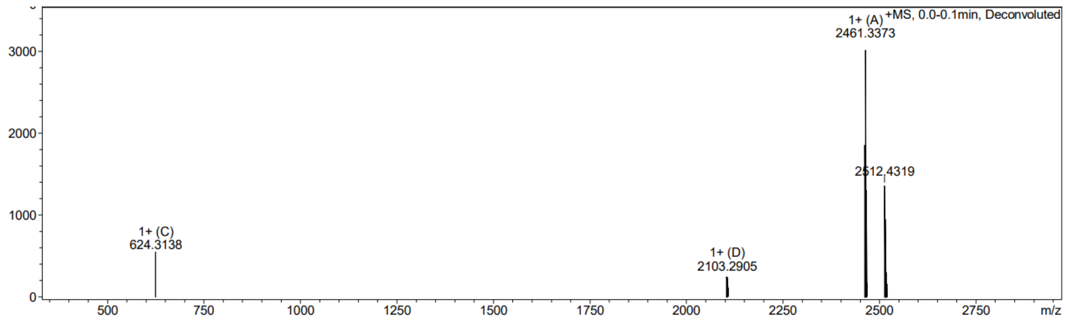

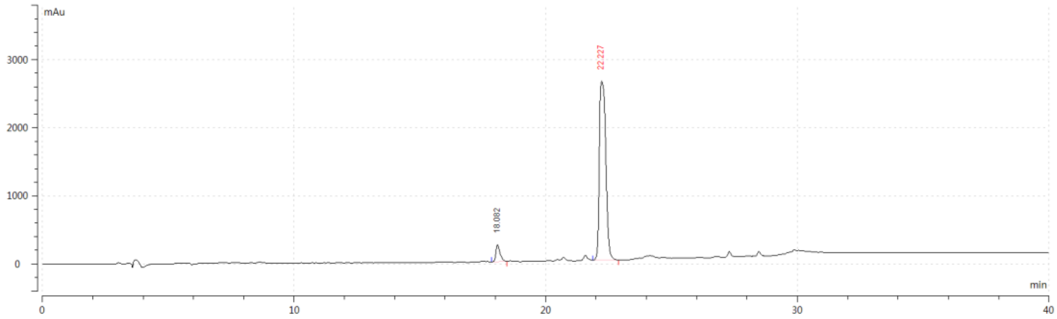

Fig. S18 Structure, HPLC analysis and MS spectrum of SA$_2$-(PEG$_2$)$_2$-K(FAM)-CAR

SA$_2$-PEG$_{2000}$-CAR: SA$_2$-K-PEG$_{2000}$-K-CARSKNKDC-OH

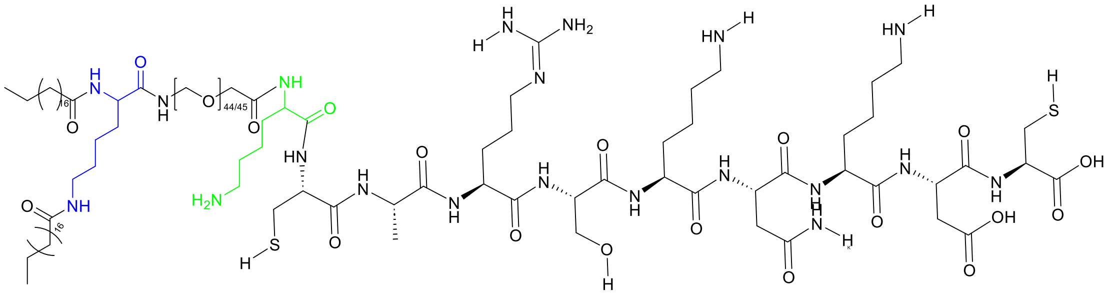

Chemical Formula: $C_{176}H_{340}N_{20}O_{63}S_2$

Exact Mass: 3806.35, M+H$^+$=3807.3486

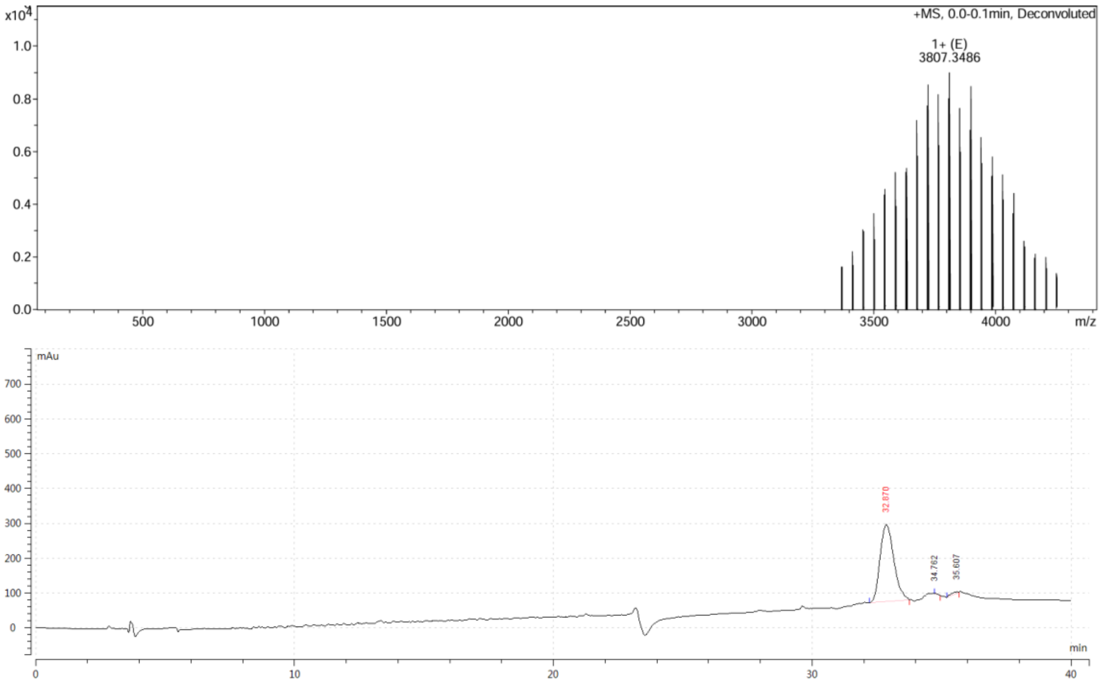

Fig. S19 Structure, HPLC analysis and MS spectrum of SA$_2$-PEG$_{2000}$-CAR

SA$_2$-PEG$_{2000}$-K(FAM)-CAR: SA$_2$-K-PEG$_{2000}$-K(FAM)-CARSKNKDC-OH

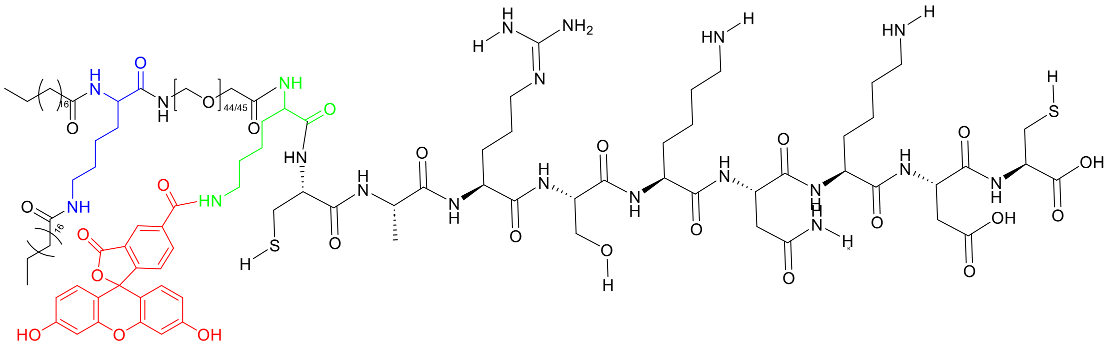

Chemical Formula: $C_{197}H_{350}N_{20}O_{69}S_2$

Exact Mass: 4164.39, M+H$^+$=4165.39

Here 4170 is only five molecular weights larger than 4165, which may be due to detector instability, but this result is still correct.

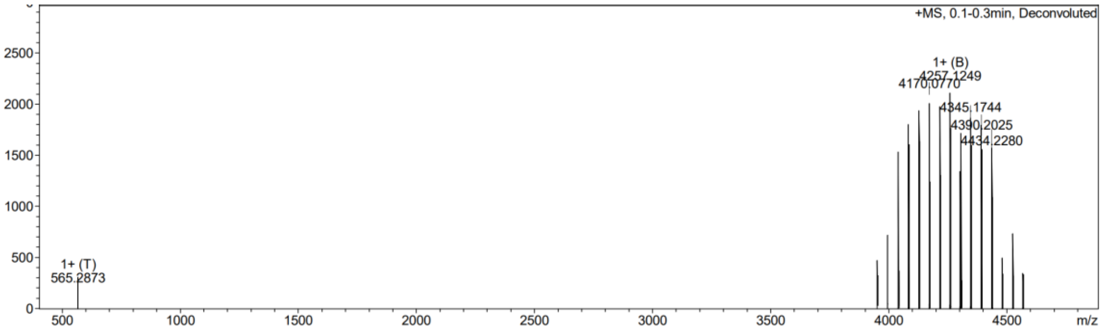

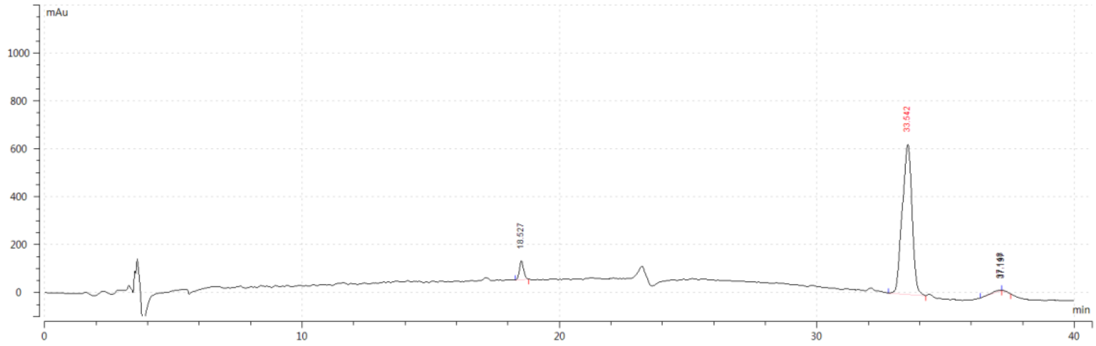

Fig. S20 Structure, HPLC analysis and MS spectrum of SA$_2$-PEG$_{2000}$-K(FAM)-CAR
